# Network changes in psychopathological symptoms following cognitive behavioral therapy for social anxiety disorder: evidence from 29 university outpatient clinics

**DOI:** 10.1186/s40359-026-05900-9

**Published:** 2026-11-10

**Authors:** Xu Wen, Julia Velten, Hanna Christiansen, Jürgen Hoyer, Tina In-Albon, Tania M. Lincoln, Wolfgang Lutz, Julian A. Rubel, Brian Schwartz, Rudolf Stark, Katja Werheid, Ulrike Willutzki, Stephan Bartholdy, Elisa-Maria Berger, Anne-Kathrin Bräscher, Thomas Forkmann, Julia A. Glombiewski, Miriam Hehlmann, Sylvia Helbig-Lang, Andrea Hermann, Christiane Hermann, Anke Kirsch, Tim Klucken, Ulrike Lueken, Tanja Michael, Patrizia Odyniec, Anya Pedersen, Andre Pittig, Babette Renneberg, Anne Katrin Risch, Almut Rudolph, Saskia Scholten, Tabea Schweden, Tobias Teismann, Antonia Vehlen, Gabriele Wilz, Jürgen Margraf

**Affiliations:** 1 Mental Health Research and Treatment Center, Faculty of Psychology, Ruhr University Bochum, Bochum, Germany; 2 Clinical Psychology and Psychotherapy, Department of Psychology and Psychotherapy, Faculty of Health, Witten Herdecke University, Witten, Germany; 3 Department of Psychology, Clinical Child and Adolescent Psychology, Philipps-University Marburg, Marburg, Germany; 4 Institute of Clinical Psychology and Psychotherapy, Technische Universitaet Dresden, Dresden, Germany; 5 Clinical Child and Adolescent Psychology and Psychotherapy, University of Mannheim, Mannheim, Germany; 6 Clinical Psychology and Psychotherapy, University of Hamburg, Hamburg, Germany; 7 Clinical Psychology and Psychotherapy, Department of Psychology, Trier University, Trier, Germany; 8 Psychotherapy Research and Clinical Psychology, Department of Psychology, Osnabrueck University, Osnabrück, Germany; 9 Department of Clinical Psychology, Psychotherapy, and Experimental Psychopathology, Johannes Gutenberg University of Mainz, Mainz, Germany; 10 Department of Psychotherapy and Systems Neuroscience, Justus-Liebig University, Gießen, Germany; 11 Department of Psychology, Bielefeld University, Bielefeld, Germany; 12 Department of Clinical Psychology and Psychotherapy, University of Greifswald, Greifswald, Germany; 13 Psychological Institute, Johannes Gutenberg University of Mainz, Mainz, Germany; 14 Department of Clinical Psychology and Psychotherapy, University of Duisburg-Essen, Essen, Germany; 15 Clinical Psychology and Psychotherapy, RPTU University Kaiserslautern-Landau, Kaiserslautern, Germany; 16 Postgraduate Clinical Training Program PTA Hamburg, University of Hamburg, Hamburg, Germany; 17 LOEWE Center DYNAMIC, University of Marburg, Marburg, Germany; 18 Department of Clinical Psychology, Justus-Liebig University Gießen, Gießen, Germany; 19 Clinical Psychology and Psychotherapy, Saarland University, Saarbrücken, Germany; 20 Clinical Psychology and Psychotherapy, Department of Psychology, Siegen University, Siegen, Germany; 21 Department of Psychology, Humboldt-Universität zu Berlin, Berlin, Germany; 22 Clinical Psychology and Psychotherapy, Department of Psychology, University of Kiel, Kiel, Germany; 23 Translational Psychotherapy, University of Göttingen, Göttingen, Germany; 24 Clinical Psychology and Psychotherapy, Freie Universitaet Berlin, Berlin, Germany; 25 Department of Counseling and Clinical Intervention, Friedrich-Schiller University Jena, Jena, Germany; 26 Clinical Psychology and Psychotherapy, Department of Psychology, University of Leipzig, Leipzig, Germany; 27 Clinical Psychology and Neuroscience, University of Hamburg, Hamburg, Germany

**Keywords:** Network analysis, Cognitive behavioral therapy, Social anxiety disorder, Network comparison.

## Abstract

While network theory posits that interactions between social anxiety symptoms perpetuate social anxiety disorder (SAD), findings on how these networks change following treatment remain inconsistent. This study used data from 29 university outpatient clinics in Germany to estimate psychopathological symptom networks for patients with SAD at pre-treatment, explore critical symptoms and connections in pre- and post-treatment networks, and identify network changes in remitted vs. non-remitted patients. By using the Brief Symptom Inventory to measure transdiagnostic symptoms in 2,392 patients with SAD, 59.8% female, *M*(*SD*)_age_ = 30.46 (10.71), we estimated six networks using Gaussian Graphical Models. Further, network comparisons were performed between patients who achieved remission (*n* = 277) and those who did not achieve remission of SAD at post-treatment (*n* = 266). In baseline networks, four themes emerged: negative self-evaluation, loneliness, problems with cognitive functioning, and fear-related experiences. The network structure and global connectivity of remitted networks were significantly changed after treatment (*M* = 0.30, *p* = .012; *S* = 20.46, *p* < .001). The global strength of non-remitted networks increased, whereas the overall network structure remained largely unchanged after treatment (*M* = 0.30, *p* = .203; *S* = 21.93, *p* = .011). This study identified four core symptom themes in networks of patients with SAD. Structural changes in these themes were associated with remission after treatment, whereas the themes remained present in patients who continued to meet the criteria for SAD. These results highlight the importance of targeting core symptom clusters and their interconnections in effective SAD treatment.

**Supplementary Information:**

The online version contains supplementary material available at https://doi.org/10.1186/s40359-026-05900-9.

## Introduction

Network perspectives offer a new way to understand mental disorders and the efficacy of treatment [1, 2]. According to network theory, the relationship of symptoms to mental disorders is from part to whole, and mental disorders emerge from causal interactions between symptoms [3]. Consistent with this theory, network studies confirmed that for most disorders, symptoms are highly interconnected [4]. Empirical network analyses have consistently demonstrated high symptom network connectivity across disorders, including depression [5], social anxiety disorder (SAD; [6]), posttraumatic stress disorder (PTSD; [7]), and obsessive-compulsive disorder [8].

SAD is characterized by persistent fear and avoidance of social situations due to a fear of being evaluated by others [9]. Individuals with SAD typically have lower income, are less likely to obtain university degrees or engage in management, technical, or professional occupations compared to those without SAD [10–12]. Cognitive behavioral therapy (CBT) has been shown to effectively treat SAD in the short and long term [13–15]. In follow-up outcome studies, treatment response and remission rates in patients with SAD six months after therapy were at 72% and 44%, respectively [13].

From a pathological perspective, network theory suggests that a high number of connections between social anxiety symptoms are more likely to produce an episode of SAD [1]. In network theory, nodes represent symptoms and edges represent correlations between symptoms. Network connectivity indicates the strength of connections between symptoms, while various centrality indices reflect the importance and influence of symptoms [1]. Intuitively, a symptom is considered central when it is strongly connected to many other symptoms, so that it may activate or maintain them [1, 16, 17] conducted a comparative analysis of network connectivity between individuals with SAD and healthy controls and discovered that individuals with SAD exhibit a densely interconnected network of fear and avoidance of social situations. High interconnectivity of symptoms is also consistent with cognitive behavioral models suggesting that cognitive biases play a critical role in activating other symptoms [18]. Centrality analysis and network comparison can be used to identify the expected influence and connectedness of a given SAD symptom with all other symptoms [19, 20]. Expected influence is an index of centrality that reflects the summed strength of a symptom’s connections to all other symptoms, taking both positive and negative associations into account, and is regarded as the most useful centrality index for psychopathology networks [16] and interventions that focus on central symptoms might be more effective in reducing the overall interconnection of the network, thereby improving treatment outcome [1, 21, 22]. For example, Rodebaugh et al., [23] first identified intervention targets depending on centrality indices in a group of patients with SAD. Then they found that changes in central symptoms were more strongly related to overall symptom change in a different group of patients with SAD who underwent various psychological treatments. Although cross-sectional comparisons suggest that clinical networks are more interconnected than healthy ones, longitudinal studies reveal paradoxical patterns. Most empirical network studies investigating the efficacy of CBT observed no changes in global network connectivity [24], while few studies reported a significant increase [25–28]. To date, post-treatment psychopathological networks remain largely unexplored. However, to better understand treatment processes, it is crucial to examine longitudinal changes in symptom networks before and after treatment, rather than relying solely on cross-sectional comparisons between clinical and healthy samples [16]. A recent study that analyzed changes in social anxiety symptom networks after Internet-based CBT [29] revealed significant connections between self-focused attention and tension, and the most influential social anxiety symptom remained a key driver post treatment.

Previous studies that explored social anxiety symptom networks in patients diagnosed with SAD [6, 29] relied on rigorously screened homogeneous samples from single research clinics. However, relying on patients from individual clinics and the exclusion of patients with any comorbid diagnoses may inflate symptom connectivity estimates and reduce ecological validity ​​[16, 30]. Further, in previous network studies, both pre-treatment and post-treatment network models were estimated using SAD-specific psychometric instruments [6, 23, 29]. More fundamentally, networks constructed from SAD-specific scales cannot detect how social anxiety symptoms interact with transdiagnostic features like depressed mood or somatic complaints, interactions that likely reflect comorbid psychopathology [31, 32]. A transdiagnostic measure such as the Brief Symptom Inventory (BSI) covers a broader range of symptoms and allows the network to capture these interactions. This is particularly relevant for the present study, because the networks are compared before and after treatment and remission is defined at the diagnostic level rather than by social anxiety symptoms alone.

Noda et al., [33] reviewed SAD-specific questionnaires such as the Liebowitz Social Anxiety Scale (LSAS; [6, 17]), the Social Interaction Anxiety Scale (SIAS), or the Social Phobia Scale (SPS; [29]) for network studies and pointed out that these tools lack coverage of some symptoms crucial for understanding the psychopathology of SAD (e.g., self-focused attention), and therefore fail to account for the impact of comorbid symptoms.

Naturalistic samples including patients with comorbidities are essential to capture common symptom interactions [32]. Further, scales such as the Brief Symptom Inventory [34] that assess transdiagnostic symptoms (e.g., sleep disturbance, concentration difficulties) may be better suited to capture treatment-related network changes, particularly for patients achieving remission. In addition, no study has yet explored the network changes in the general psychopathological symptom network of patients who no longer meet the SAD diagnostic criteria after treatment. Leveraging treatment data from 29 university outpatient clinics collaborating to facilitate naturalistic effectiveness research ​[15, 35–37], th​is study focused on the following goals: Firstly, this study aimed to estimate two distinct psychopathological networks at baseline: one for all patients diagnosed with SAD and one for a subsample of patients with SAD without comorbid mental health diagnoses. This was done in order to examine influential symptoms and connections in patients with SAD. Secondly, this study used network comparison tests (NCTs) to identify significant changes in symptom centrality, as well as any modifications or restructuring of the network, by comparing the pre- and post-treatment networks of patients who had achieved remission of SAD. Thirdly, this study investigated symptom persistence by comparing pre- and post-treatment networks in patients who did not remit, in order to identify stable central symptoms and the potential reasons for persistent connectivity. On the basis of previous findings, we expected that psychopathological symptom networks would change from pre- to post-treatment. Because previous studies reported inconsistent findings and no study had examined the networks of patients who no longer met the SAD criteria, no directional hypotheses were formulated for the comparison between remitted and non-remitted patients, and this comparison was exploratory.

## Method

### Participants

To establish baseline psychopathological symptom networks for a naturalistic sample of patients with SAD, a dataset of 2,392 patients (59.8% female; *M*_age_ = 30.46, *SD* = 10.71), treated in 29 collaborating university outpatient clinics between 2018 and 2024, was used (see Table 1 for an overview on the demographic characteristics). Before treatment, patients had an average of 2.32 (*SD* = 0.99) diagnoses. The most common comorbid diagnoses at baseline were mood disorders such as depression (Moderate depressive episode, *n* = 232; Recurrent depressive disorder, current episode moderate, *n* = 535; Recurrent depressive disorder, currently in remission, *n* = 128; Dysthymia, *n* = 220) and other anxiety or stress-related disorders (Agoraphobia, *n* = 110; PTSD, *n* = 125). All patients provided written informed consent for their anonymized data to be used in research projects. The study protocol was approved by the Ethics Committee of the Faculty of Psychology at Ruhr University Bochum (Reference No. 228).

**Table 1:** The sociodemographic description of the sample

	Total (*n* = 2392, valid%)
School education	
Still in school	67 (3.6%)
No degree	24 (1.3%)
Basic school certificate	147 (7.8%)
Intermediate school certificate	368 (19.5%)
High School Degree	1255 (66.5%)
Other	26 (1.4%)
**Education**	
Working towards degree	453 (24.3%)
No degree	183 (9.8%)
Vocational training	409 (21.9%)
College or university degree	382 (20.5%)
Other	440 (23.6%)

### Measures

#### Clinical diagnoses

Clinical diagnoses were determined with structured diagnostic interviews at each clinic, namely the Structured Clinical Interview (SCID; [38]) and the Diagnostic Interview for Mental Disorders (DIPS; [37, 39, 40]), which assess ICD-10, DSM-IV or DSM-5 criteria. The type of diagnostic assessment was recorded for every patient; at baseline, 85.7% (2050 of 2392) of the diagnoses were based on a complete structured or standardized interview. Up to five diagnoses (i.e., index diagnosis plus four additional comorbid diagnoses) were recorded.

#### Psychopathological symptom measures

The Brief Symptom Inventory (BSI; [34]) measures 53 psychopathological symptoms that are rated on a 5-point Likert scale from 0 (*not at all*) to 4 (*extremely*; see Table 2 for a list of symptoms and the respective abbreviated node names). The BSI is a widely used questionnaire designed to measure psychological distress and symptoms of mental disorders. In the present study, internal consistency at baseline was excellent (α = 0.95).

**Table 2:** Nodes and their represented psychopathological symptoms based on items of the Brief Symptom Inventory

Node	Symptom representation	Node	Symptom representation
Argue	Getting into frequent arguments	Lonely	Feeling lonely
Alone	Feeling lonely even when you are with people	MemP	Trouble remembering things
Avoid	Having to avoid certain things, places, or activities because they frighten you	MindC	The idea that someone else can control your thoughts
Blame	Feeling others are to blame for most of your troubles	MindP	The idea that something is wrong with your mind
Blank	Your mind going blank	Nausea	Nausea or upset stomach
Blue	Feeling blue	Nerv	Nervousness or shakiness inside
Break	Having urges to break or smash things	NoApp	Poor appetite
Breath	Trouble getting your breath	NoCredit	Others not giving you proper credit for your achievements
Compul	Having to check and double check what you do	NotClose	Never feeling close to another person
ConcenP	Trouble concentrating	NoHope	Feeling hopeless about the future
DeathT	Thoughts about death and dying	NoInitia	Difficulty in starting
Decision	Difficulty making decisions	NoInte	Feeling no interest in things
Disliked	Feeling that people are unfriendly or dislike you	NoWorth	Feeling of worthlessness
Distrust	Feeling that most people cannot be trusted	Numb	Numbness or tingling in parts of your body
Dizzi	Faintness or dizziness	Panic	Spells of terror or panic
Exploit	Feeling that people will take advantage of you if you let them	PainChest	Pains in the heart or chest
FearAlo	Feeling nervous when you are left alone	Punish	The idea that you should be punished for your sins
FearCro	Feeling uneasy in crowds	Restless	Feeling so restless you couldn’t sit still
Fearful	Feeling fearful	Shy	Feeling very self-conscious with others
FearOpen	Feeling afraid in open spaces	Sleep	Trouble falling asleep
FearTran	Feeling afraid to travel on buses, subways, or trains	SuiciT	Thoughts of ending your life
FearSud	Suddenly scared for no reason	Temper	Temper outbursts that you could not control
Flashes	Hot or cold spells	Tension	Feeling tense or keyed up
Guilt	Feelings of guilt	Violence	Having urges to beat, injure, or harm someone
Hurt	Your feelings being easily hurt	Watched	Feeling of being watched or talked about by others
Inferio	Feeling inferior to others	Weak	Feeling weak in parts of your body
Irrit	Feeling easily annoyed or irritated		

Note. “Node” is the abbreviated node name for each symptom in the network, and “Symptom representation” indicates the psychopathological symptom being measured

### Procedure

All patients attended the university outpatient clinics of the KODAP network and received disorder-specific CBT on an outpatient basis. Treatment was delivered individually and was provided by licensed clinical psychologists and by therapists in postgraduate training, who had completed at least a master’s degree in clinical psychology and 1.5 years of clinical experience. Therapists in training attended regular supervision, one for every fourth treatment session, with a licensed CBT supervisor. Treatments generally followed published CBT guidelines for the respective disorder but were less standardized than in randomized controlled trials. The German health insurance system routinely covers 24 to 60 sessions; the average number of sessions in the KODAP sample after the diagnostic phase was about 40 (SD = 22; [15]). All clinics belong to the same research network and followed the same training, supervision and documentation standards, so that the treatment procedures were largely comparable across the 29 clinics.

To investigate the psychopathological symptom networks of patients with SAD, we used the full dataset of 2,392 patients diagnosed with SAD at baseline. To further estimate symptom networks unaffected by comorbid mental disorders, we selected 317 patients with SAD without additional comorbid diagnoses at baseline (13.25% of the original dataset, 60.1% female; *M*_age_ = 28.03 years old, *SD* = 8.64). To identify psychopathological symptom network changes after treatment, we applied sequential exclusion criteria to the original dataset: First, we excluded 90 patients with four or more diagnoses in total, that is, the index diagnosis plus at least three comorbid diagnoses, from the original dataset to reduce transdiagnostic symptom overlap and increase specificity to SAD pathology. Because the study addresses the course of SAD, and because such complex comorbidity profiles can reduce the stability of the estimated networks, this criterion affected only a small part of the sample (3.8%). Second, data were checked for longitudinal completeness. To enable network comparison tests, 1,449 patients lacking post-treatment BSI scores and 310 patients without post-treatment diagnostic interviews were excluded, yielding a final analytic sample of 543 patients with complete pre-post symptom assessments and diagnostic evaluations. The post-treatment diagnostic assessment was conducted at the end of treatment or, if no assessment was available at that point, at the last available assessment after baseline. Remission was defined as no longer meeting the diagnostic criteria for SAD at this assessment, which was part of the routine procedure of the respective clinic and was not conducted by independent assessors; residual symptoms below the diagnostic threshold were not taken into account. These patients were divided into two groups: patients who achieved SAD remission at post-treatment (*n* = 277) and patients who did not (*n* = 266). Their descriptive characteristics are reported in the Results.

### Data analysis

Six psychopathological networks were estimated in this study. All six symptom networks were estimated using Gaussian Graphical Models (GGM), where nodes represented individual psychological symptoms and edges corresponded to regularized partial correlations between symptoms after accounting for all other variables in the model, and all correlations were based on Spearman correlation matrices [41]. To enhance the accuracy of network connections, we applied the graphical LASSO algorithm with a tuning parameter that balanced network sparsity and connection retention, reducing false positive associations while preserving clinically relevant relationships [42]. Visual representations of networks were generated using the Fruchterman-Reingold algorithm, which positions strongly interconnected symptoms closer to the network center, with line thickness reflecting correlation magnitude [43]. All computations were implemented through the qgraph package (Version 1.6.9) in R. The centrality of nodes was evaluated using Expected Influence (EI), a metric that considers both positive and negative connection weights [4]. This approach proves particularly valuable when analyzing symptom networks with inhibitory relationships and shows stronger predictive power for longitudinal clinical outcomes compared to other centrality measures [4, 16]. Network stability was assessed through bootstrap resampling methods. One thousand bootstrap samples were used to examine the consistency of edge weights and centrality indices (including 95% confidence intervals for edge weights), a case-dropping bootstrap with 1000 resamples was used to obtain the centrality stability coefficient, and statistical comparisons between edges were conducted through pairwise difference tests. For network comparisons between pre-treatment and post-treatment states, the network comparison test was employed with 10,000 permutations to evaluate significant changes in network properties across different networks (van Borkulo et al., 2023). Because the pre- and post-treatment networks are based on repeated observations from the same participants, the comparison was implemented as a dependent (paired) permutation test. The implementation of the network comparison test for dependent data is not yet formally validated, as stated in the documentation of the R package, and the node-level results are therefore interpreted as exploratory. Specifically, permutation-based comparisons were carried out to compare network invariance, global strength invariance, changes in node expected influence, and modifications in edge weights across different treatment phases. To examine whether the symptom themes described below are also supported quantitatively, a community detection analysis was additionally performed on the baseline networks using the walktrap algorithm, which groups nodes into communities on the basis of the random walk structure of the network [44]. The four symptom themes reported below were identified conceptually, on the basis of the pattern of the strongest edges and of established cognitive behavioral models of SAD, and the community detection analysis was used as a descriptive check of this grouping.

## Results

**Fig. 1:**
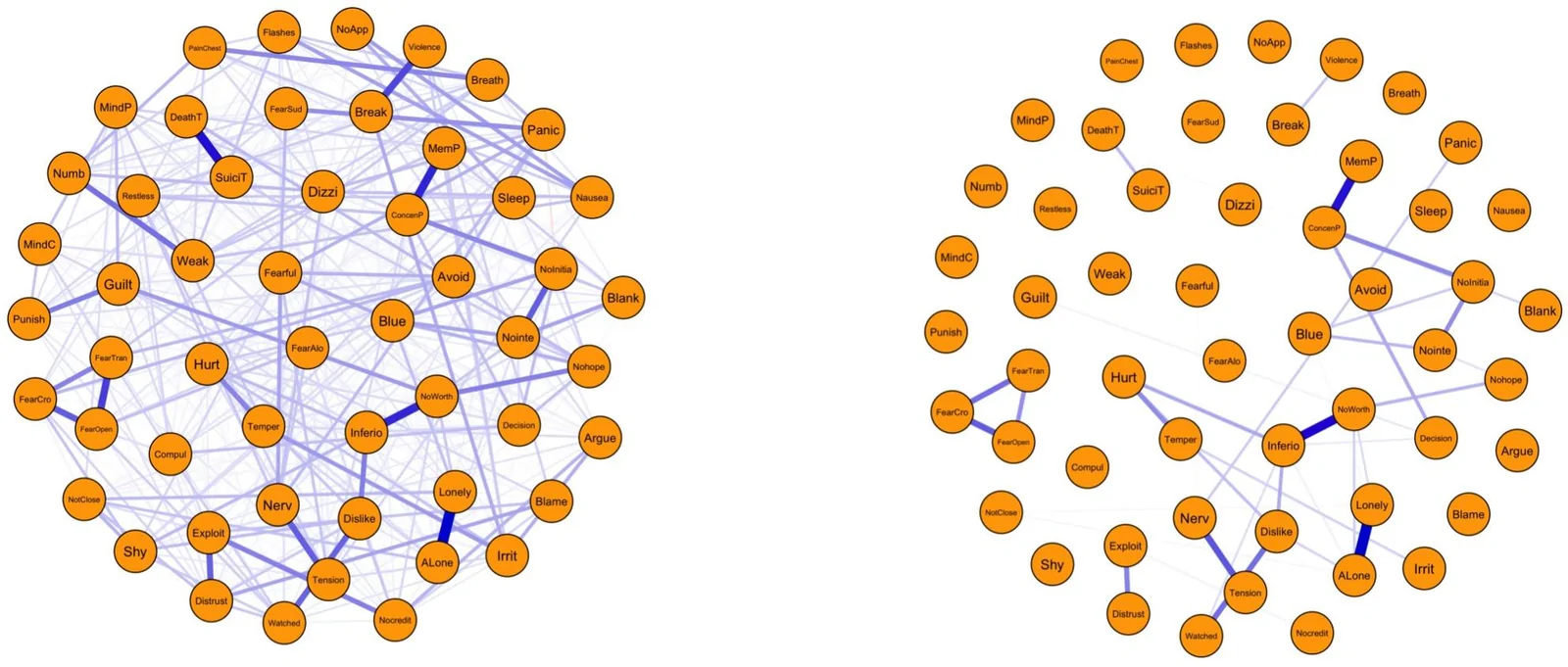
Psychopathological networks for patients with social anxiety disorder (left, *n* = 2392) and without comorbid diagnoses (right, *n* = 317) at baseline. Note. Nodes represent symptoms, blue edges represent positive partial correlations between symptoms while red edges represent negative partial correlations. Parameter: cut = 0, minimum = 0

**Fig. 2:**
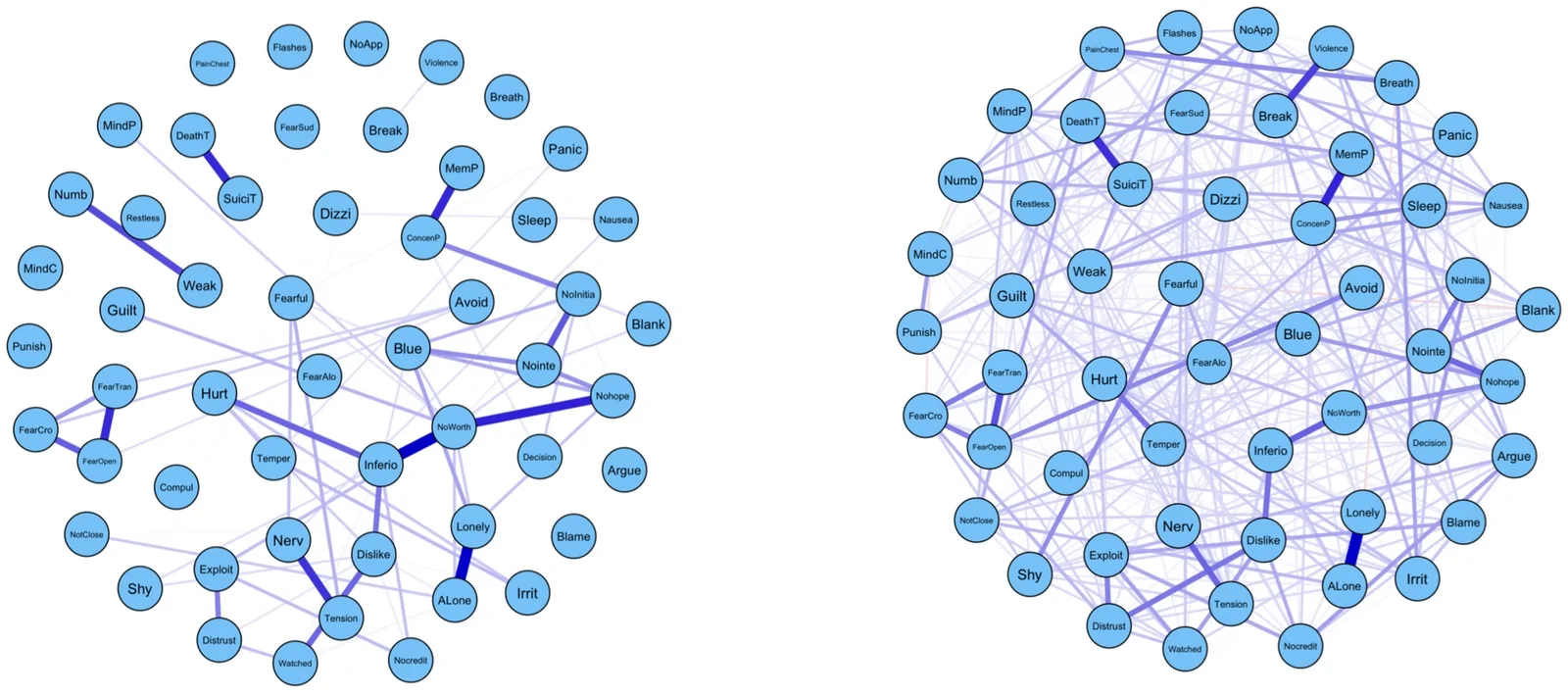
Psychopathological networks at baseline (left) and at post-treatment (right) for patients who achieved SAD remission after treatment (*n* = 277). Note. Nodes represent symptoms, blue edges represent positive partial correlations between symptoms while red edges represent negative partial correlations. Parameter: cut = 0, minimum = 0

**Fig. 3:**
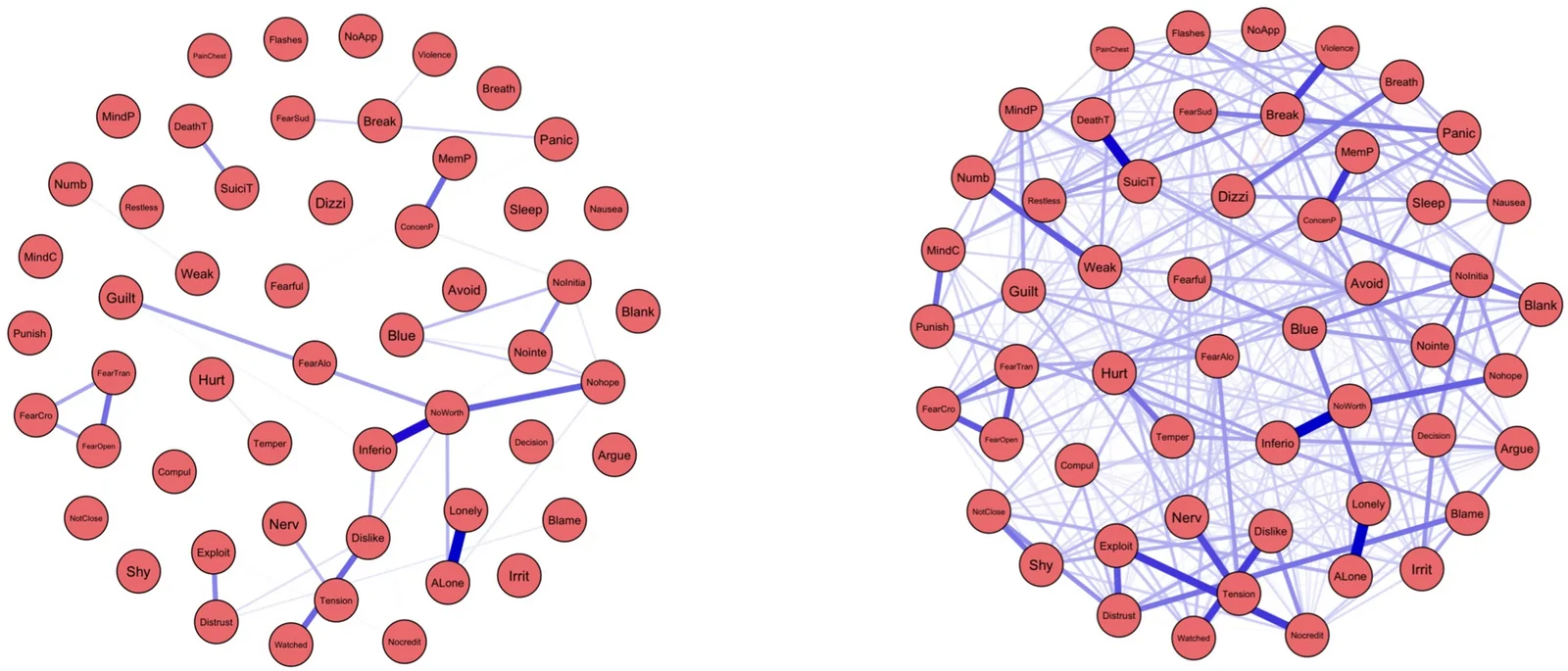
Psychopathological networks at baseline (left) and at post-treatment (right) for patients who did not achieve SAD remission after treatment (*n* = 266). Note. Nodes represent symptoms, blue edges represent positive partial correlations between symptoms while red edges represent negative partial correlations.Parameter: cut = 0, minimum = 0

Figures 1, 2 and 3 show the psychopathological symptom networks for patients with SAD.

The final analytic sample comprised 543 patients, of whom 277 achieved remission of SAD and 266 did not. The remitted patients had a mean age of 29.68 years (*SD* = 10.05) and 63.5% were female, with a mean of 2.04 diagnoses (*SD* = 0.69) at baseline and 0.62 at post-treatment (*SD* = 0.70). The non-remitted patients had a mean age of 30.82 years (*SD* = 11.53) and 57.9% were female, with a mean of 2.01 diagnoses (*SD* = 0.68) at baseline and 1.73 at post-treatment (*SD* = 0.73). The most common comorbid diagnoses at baseline were mood disorders (remitted: moderate depressive episode *n* = 18, recurrent depressive disorder with a current moderate episode *n* = 57, dysthymia *n* = 33; non-remitted: *n* = 16, *n* = 56 and *n* = 21, respectively). Symptom severity decreased from pre- to post-treatment in both groups. In the remitted patients the mean Global Severity Index of the BSI decreased from 1.13 (*SD* = 0.57) to 0.62 (*SD* = 0.56), *t*(276) = 15.74, *p* < .001; in the non-remitted patients it decreased from 1.17 (*SD* = 0.58) to 0.76 (*SD* = 0.56), *t*(265) = 12.82, *p* < .001. Descriptive statistics for the individual symptoms discussed in the text are provided in Table C3.

To assess possible selective attrition, the 543 included patients were compared with the excluded patients on baseline characteristics. The two groups did not differ in age (30.24 vs. 30.53 years, *p* = .58) or in the proportion of women (60.8% vs. 59.8%), but the excluded patients had more comorbid diagnoses (2.40 vs. 2.03, *p* < .001) and a higher baseline symptom severity (Global Severity Index 1.29 vs. 1.15, *p* < .001).

### Network stability test

Network stability was evaluated through 1,000 bootstrap iterations, with edge weight stability and centrality index consistency quantified across subgroups. The bootstrap tests of all six psychopathological networks revealed that the structural stability in all networks was robust for medium to strong edges (edge weight more than 0.1), which allows for further interpretation of significant edges (for bootstrap results, see bootstrapped stability test figures in Appendix A. Supplementary Material). The centrality stability coefficient for expected influence was 0.36 for the pre-treatment networks of both groups and 0.05 and 0.00 for the post-treatment networks of the remitted and the non-remitted patients, which indicates that the centrality estimates, in particular after treatment, should be interpreted with caution.

Based on the outcomes of the bootstrapped edge difference test (*α* = .05) for all psychopathological symptom networks, some edges exhibited strong interconnected strength across all networks. The edges connecting the symptoms “Feeling lonely” and “Feeling lonely even when you are with people”, the edges between the symptoms “Feeling of worthlessness” and “Feeling inferior to others”, and the edge between the symptoms “Trouble concentrating” and “Trouble.

remembering things” were stronger than most of the other edges across all networks. Additionally, the triangular edges connecting the three fears “Feeling afraid in open spaces”, “Feeling uneasy in crowds”, and “Feeling afraid to travel on buses, subways, or trains”, demonstrated higher strength than most other edges (for details, see the bootstrapped edge difference test figures in Appendix B. Supplementary Material).

Several remarkably strong edges were found in the networks for both patients who achieved SAD remission at post-treatment and those who did not. In the baseline network of patients who later achieved remission, the strongest edges were those between “Feeling of worthlessness” and “Feeling inferior to others” (*w* = 0.28, 95% CI [0.23, 0.40]), between “Feeling lonely” and “Feeling lonely even when you are with people” (*w* = 0.27, 95% CI [0.24, 0.46]), and between “Numbness or tingling in parts of your body” and “Feeling weak in parts of your body” (*w* = 0.17, 95% CI [0.11, 0.41]). For the post-treatment networks of both remitted and non-remitted patients, the edge between “Thoughts of ending your life” and “Thoughts about death and dying” was among the strongest (remitted: *w* = 0.36, 95% CI [0.21, 0.43]; non-remitted: *w* = 0.40, 95% CI [0.26, 0.47]). For the post-treatment network of patients who achieved remission, the edge between “Having urges to break or smash things” and “Having urges to beat, injure, or harm someone” was particularly strong (*w* = 0.33, 95% CI [0.16, 0.43]). For the post-treatment network of non-remitted patients, the edge between “Others not giving you proper credit for your achievements” and “Feeling that people will take advantage of you if you let them” was strong (*w* = 0.30, 95% CI [0.16, 0.37]). The numerical values and confidence intervals of all reported edges are provided in Table C1.

### Centrality results of the network

**Fig. 4:**
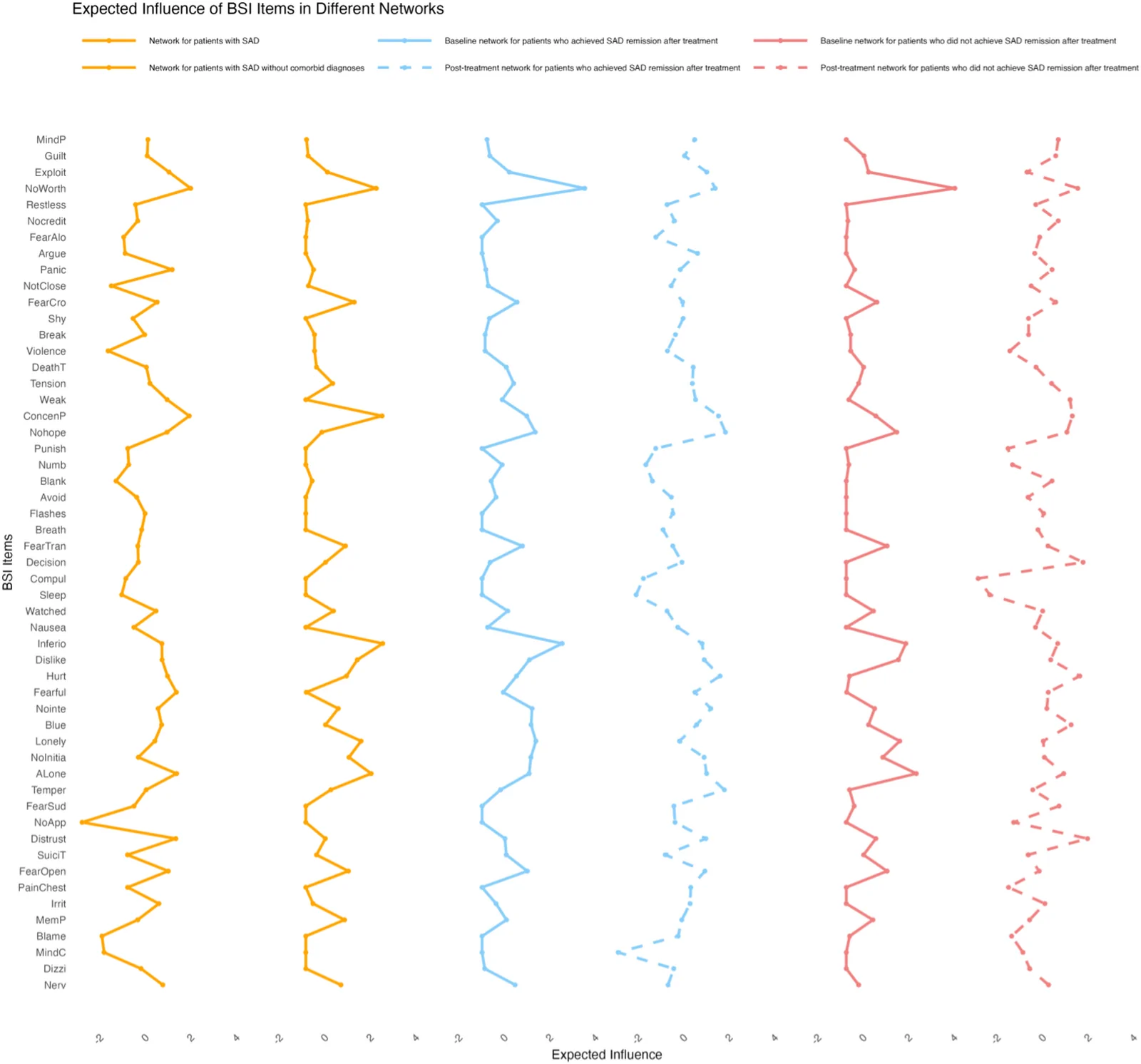
The expected influence results of six networks. Note. The lines from left to right are baseline network for patients with SAD, baseline network for patients with SAD without additional comorbidity, baseline network for patients who achieved remission from SAD at post-treatment, post-treatment network for remitted patients, baseline network for patients who do not achieve remission from SAD at post-treatment, post-treatment network for non-remitted patients. Expected Influences are shown as standardized z-scores. The two networks of each comparison are shown in the same colour, that is, orange for the two baseline networks, light blue for the networks of patients who achieved remission, and light coral for the networks of patients who did not achieve remission, and solid lines indicate baseline and pre-treatment networks whereas dashed lines indicate post-treatment networks

The specific Expected Influence (EI) for each node in the six networks is presented in Fig. 4. Interesting patterns were observed in the overall morphological characteristics of EI: Three baseline networks (baseline network of patients with SAD without comorbidities, baseline networks of patients who later achieved remission, and baseline networks of patients who later did not achieve remission) showed highly similar EI characteristics, which significantly diverged from those of post-treatment networks. Specifically, for the baseline networks for patients who later achieved remission as well as those who did not, the EI figure showed that the nodes “Feeling of worthlessness” and “Feeling inferior to others” exhibited extremely strong EI, and the nodes “Feeling lonely”, “Feeling lonely even when you are with people”, and “Trouble concentrating” also showed strong EI (See Expected Influence for each node in Fig. 4). The three ‘Fear’ nodes (i.e., “Feeling uneasy in crowds”, “Feeling afraid to travel on buses, subways, or trains”, and “Feeling afraid in open spaces”) exhibited high EI in pre-treatment networks of both remitted and non-remitted patients. In the post-treatment network for patients who achieved remission, the EI figure showed that node “Feeling hopeless with the future” and node “Temper outbursts that you could not control” emerged as the highest EI nodes. In contrast, in the post-treatment network for non-remitted patients, node “Feeling that most people cannot be trusted”, node “Difficulty making decisions”, and node “Feeling of worthlessness” were among the nodes with the highest EI, and the relative position of “Feeling of worthlessness” decreased from pre- to post-treatment. The expected influence of every node before and after treatment, together with the statistical comparison, is reported in Table C2. Because the centrality stability coefficient was low for the post-treatment networks, the node-level centrality results should be interpreted as exploratory.

### Network comparison test

The network comparison test indicated substantial changes in network structure, global strength, and EI of nodes between the compared networks. For patients who achieved remission from SAD at post-treatment, the NCT revealed significant changes in both network structure and global strength (Network invariance test: M = 0.30, *p* = .012; Global strength invariance test: S = 20.46, *p* < .001). Global strength is the sum of all absolute edge weights in a network and increased from 4.73 before treatment to 25.18 after treatment in these patients. Subsequent comparisons of nodes indicated that all nodes exhibited significant changes in EI (all False Discovery Rate-adjusted *p* < .05). The directional statements below refer to the standardized expected influence, that is, the relative position of a node within its network, as shown in Fig. 4. According to the EI figures of networks for patients who achieved SAD remission after treatment, the node “Feeling of worthlessness” and node “Feeling inferior to others” exhibited marked decreases in their EI. Meanwhile, the node “Feeling lonely”, node “Feeling uneasy in crowds”, and node “Feeling afraid to travel on buses, subways, or trains” also exhibited significant decrease in their EI. However, several nodes including “Getting into frequent arguments”, “Feeling hopeless about the future”, “Difficulty making decisions”, “Temper outbursts that you could not control”, and “Feeling that most people cannot be trusted” increased their EI in post-treatment network.

For patients who did *not* achieve SAD remission at post-treatment, the network invariance test indicated no significant network structure changes after treatment (*M* = 0.30, *p* = .203). The global strength invariance test showed that the global strength of the post-treatment network of these patients was significantly stronger than that of their baseline network (S = 21.93, *p* = .011), corresponding to an increase from 2.60 to 24.53. Almost no significant changes in EI were observed; after the False Discovery Rate correction, only 3 of the 53 nodes remained significant (all other ps ≥ 0.050). Regarding the two baseline networks for patients with SAD and with SAD without additional comorbidity, the results of the network invariance test and global strength invariance test were not significant (*M* = 0.36, *p* = .069; *S* = 21.50, *p* = .208).

## Discussion

This study examined psychopathological symptom networks in patients diagnosed with SAD before and after receiving CBT across 29 university outpatient clinics in Germany. Six distinct networks were estimated on the basis of psychopathological symptoms to allow comparisons of network structure and changes between baseline and post-treatment assessments in patients who had achieved remission and those who had retained their SAD diagnosis at post-treatment. In baseline networks, four symptom themes including negative self-evaluation, loneliness, problems with cognitive functioning, and fear-related experiences were identified. The structure and global connectivity of psychopathological networks for patients who achieved remission of SAD were changed, and the core connections between self-evaluation symptoms were significantly reduced after treatment. Meanwhile, the network structure of four symptom themes was still present in networks of non-remitted patients.

This study bridges a critical gap in understanding the symptom networks of patients with SAD as prior network studies limited their analyses to disorder-specific measures such as the LSAS or SIAS ([6] b; [29]). In line with previous studies which used SAD-specific scales to estimate social anxiety networks, we found similarly densely interconnected symptom networks at baseline using a measure that assessed a broad range of psychopathological symptoms ([6] b; [29, 33]). Identifying influential symptoms and their connections in psychopathological symptom networks can improve our understanding of the psychopathology of SAD [1, 3, 16].

In this study, influential symptoms and connections in baseline networks reflected four themes. These themes were identified conceptually, based on the pattern of the strongest edges and on established cognitive behavioral models of SAD. A walktrap community detection analysis of the baseline networks broadly corresponded to this grouping: the fear-related items formed one community, the cognitive and depressive items another, the self-evaluation items clustered with the loneliness items, and the suicide-related items formed a separate community. Negative self-evaluation included the highly central symptom of feelings of worthlessness and its connections to other symptoms related to self-evaluation such as feeling inferior to others. Loneliness included this core symptom and connections to two other loneliness-related symptoms. Problems with cognitive functioning included the core symptom of concentration difficulties and connections with other cognitive symptoms such as troubles remembering things. Lastly, fear-related experiences included symptoms of fear such as fear of open places or of using public transport and the connections between these symptoms. Although patients without comorbid diagnoses had a baseline network with fewer connections among symptoms, the same four themes were observed.

The identification of these themes invites closer consideration of how they connect to established cognitive behavioral models and prior research on the development and maintenance of SAD. The most influential theme in the network, negative self-evaluation, is also identified as a central role in the development and maintenance of SAD [45, 46] and is a key component of many cognitive behavioral models (e.g., Clark and Wells, [18], Heimberg, [47], Rapee, [48]. Further, loneliness is known to heighten sensitivity to social threats and to impair executive functioning, sleep as well as mental and physical well-being [49]. Previous studies indicated that impairments in both attention allocation (e.g., the ability to disengage attentional resources from emotional stimuli) and memory (e.g., the ability to memorize emotional stimuli) might be root causes for why patients with SAD exhibit attentional and memory biases [50, 51]. Further, attentional biases to external threat and memory biases play critical roles in the development and persistence of social anxiety symptoms [18, 45, 48, 52]. Lastly, the close interconnections among fear-related symptoms may reflect the intense fearful experiences that characterizes SAD. As Heimberg et al., [47] noted, individuals with SAD experience fear not only in actual social situations but also in anticipation of entering public contexts where external evaluation is likely, or after a social situation [45].

The diversity of the four types of core symptoms identified in our study is consistent with a previous scoping review that highlighted the need to examine the network structure of SAD in its full complexity, including emotional, behavioral, cognitive, physical, and attentional symptoms, as well as symptoms related to comorbid disorders [33]. Therefore, constructing networks of psychopathological symptoms to identify core symptoms is highly informative. This approach not only supports existing etiological models but also facilitates the identification of key symptom clusters within the broader complexity of SAD psychopathology [18, 48, 52].

Additionally, an interesting finding was that symptoms related to depressive mood and suicidal ideation (e.g., thoughts about death and ending your life) were also significantly connected in the networks of patients without comorbid diagnoses. This finding indicates that suicidal ideation can negatively affect patients with SAD even if they are not diagnosed with comorbid depressive disorders. This finding is also consistent with the results of longitudinal SAD network studies, which have shown that depressive symptoms form the strongest connections within combined social anxiety–depression networks at later stages of therapy [53, 54].

Regarding the changes after treatment, a comparison of networks revealed both remitted and non-remitted patients with SAD exhibited a more densely interconnected network after CBT, but significant structural changes were only found in patients who achieved SAD remission after treatment. Specifically, while both remitted and non-remitted patients showed an emergence of connections between symptoms that fell outside the four core symptom themes, only remitted patients also showed reductions in connections between symptoms within these themes. This led to significant changes in the overall structure of the network. The increase in global network strength is consistent with prior treatment studies that reported an increase in global network connectivity after CBT [25, 27, 28]. This increase should, however, be interpreted with caution. Indices such as global strength depend on how the network is estimated and on the level and the variability of symptoms, and the post-treatment assessments in our data were characterized by lower symptom levels and lower item variability. The increase was also present in both groups and therefore does not distinguish remission from non-remission. We therefore do not interpret it as a direct indicator of greater psychopathology.

For the changes within the four main symptom themes, in the networks of patients who achieved remission, the central influence of all four themes was reduced. Notably, we found that the symptoms relating to feelings of worthlessness and inferiority, which were identified as the most critical symptoms and the most relevant core connection in all pre-treatment networks, showed marked attenuation in expected influence only in the post-treatment network of remitted patients. This attenuation is consistent with the cognitive model of SAD and may indicate that, after CBT, patients are less likely to internalize negative evaluations of their performance as reflections of their self-worth. This may interrupt the cascade of behavioral, physical, and cognitive symptoms of anxiety that was present before treatment [47, 48]. Because these mechanisms were not directly measured in the present study, this interpretation remains tentative. Such cognitive restructuring responds to the observation in previous research that self-focused cognition remained unexplored in earlier network studies [33], and re-emphasizes that self-focused evaluations constitute a key psychopathological mechanism in SAD [45, 46, 55]. Other critical symptoms shifted significantly as well, including those between different aspects of loneliness and between difficulties with concentration and memory. These changes suggest that treatment was associated both with a reduction of individual symptoms and with a reorganization of the psychopathological symptom network [3, 56].

For patients who were still diagnosed with SAD following treatment, the influence of core symptoms did not decrease significantly, and the four symptom themes remained unchanged. The network of these patients did not show any notable structural changes after treatment, such as a change in the core symptom feelings of worthlessness and inferiority. This may explain why patients did not achieve remission, as they may still experience in feelings of internalized worthlessness and inferiority, be unable to stop the closely connected cascade mentioned previously ([3]; [56]). Moreover, the remaining influential role of cognitive difficulties may suggest that cognitive biases were less responsive to change, so that patients continued to allocate attention to perceived flaws in their ability to concentrate or remember. Because these processes were not directly measured, this interpretation is tentative. This, in turn, may contribute to the persistent experience of social anxiety. In patients who did not remit, the ranking order of the most influential symptoms underwent only minor adjustments. However, these limited changes may have been insufficient to be associated with remission [18, 45, 57].

Taken together, these findings suggest that for patients still meeting SAD criteria, explicitly targeting the four symptom clusters identified in this study, might be beneficial. For example, for patients who appear to determine their self-worth by comparing themselves to others (i.e., those who have a strong connection between feelings of inferiority and worthlessness), discussing this in therapy could help them to restructure their symptoms in a way that is associated with less distressing social anxiety. A combination of therapeutic methods focusing on cognitive restructuring supplemented by cognitive bias modification trainings for attention and memory might also increase the chance of remission [51, 58].

Finally, this study advances the literature on psychopathological symptom networks of patients with SAD in multiple ways. The patient sample used for network estimation in this study was representative of the care provided in university outpatient clinics in Germany, ensuring ecological validity. Moreover, diagnoses were based on structured clinical interviews at baseline and at post-treatment, which supports the reliability of the clinical classification used to define the groups.

However, the exclusion of patients with incomplete data limits the generalizability of findings. Although excluding patients was necessary to allow for comparisons between pre- and post-treatment networks, it is important to consider that these patients may have characteristics that distinguish them from those with complete data. For example, they may have been less willing or able to comply with psychological assessment procedures [30]. Additionally, the study lacks mid-treatment and longer-term follow-up data. A longitudinal network analysis based on continuous measurement data collected during treatment could reveal changes in the networks at different stages of treatment [16, 44]. Meanwhile, long-term follow-up data can provide insight into the sustained efficacy of treatment and help predict treatment dropout [59]. Finally, this study focused on a general measure of psychopathology and did not include disorder-specific scales. Combining both specific and transdiagnostic measures could contribute to a better understanding of how the connections between symptoms related to mood or depression and SAD-symptoms are affected by treatment. Several further limitations should be noted. First, data were pooled across 29 outpatient clinics, and although all clinics followed published CBT guidelines, treatment was delivered as individualized routine care, so that some variation between clinics cannot be excluded; in addition, the diagnostic systems differed between centers, which may have introduced variability into the group classification. Second, the networks at each time point are cross-sectional, and the pre- and post-treatment networks are based on repeated observations from the same participants; the implementation of the network comparison test for dependent data is not yet formally validated, and the node-level results should therefore be regarded as exploratory. Third, the centrality stability coefficient was low for the post-treatment networks, which limits the interpretation of individual central symptoms. Fourth, some of the strongest edges connect items with highly similar content, and item redundancy may therefore have contributed to these associations. Fifth, the patients who were excluded had more comorbid diagnoses and a higher baseline symptom severity than those who were included, which suggests selective attrition.

## Conclusions

Through a network perspective, we examined the complex relationships among psychopathological symptoms in patients with social anxiety disorder (SAD), both with and without comorbid mental health diagnoses. Four symptom themes emerged in baseline networks: negative self-evaluation, loneliness, problems with cognitive functioning, and fear-related experiences. Analyses of pre- and post-treatment networks in patients who did and did not achieve remission offered new insights into SAD and potential treatment targets. Remitted patients showed a reorganization of the network, with the largest relative decreases in the central influence of the self-evaluation symptoms. In contrast, non-remitted patients showed little change in the overall network structure. Taken together, these findings suggest that network restructuring was associated with remission following CBT rather than merely reflecting a reduction in symptoms. Because network indices are sensitive to the estimation procedure and to symptom levels, and because the centrality estimates were less stable after treatment, these conclusions remain tentative. From a clinical perspective, targeting the core symptom themes, especially those related to self-evaluation, may increase the likelihood of remission, thus supporting the value of network analysis in guiding SAD treatment.

## Supplementary information

Below is the link to the electronic supplementary material.

Supplementary Material 1

Supplementary Material 2

Supplementary Material 3

## Data Availability

The data that support the findings of this study are not publicly available due to an agreement between the cooperating outpatient clinics but are available from the corresponding author [XW, wen.xu@rub.de] upon reasonable request.
