# Supplementary material for: Network changes in psychopathological symptoms following cognitive behavioral therapy for social anxiety disorder: evidence from 29 university outpatient clinics
Source: BMC Psychol. 2026 Nov 10;14:1627. doi: 10.1186/s40359-026-05900-9 (PMC13750052; doi:10.1186/s40359-026-05900-9)
Supplement: Supplementary file 1 [file 40359_2026_5900_MOESM1_ESM.pdf]

Appendix C. Supplementary data

Table C1 The eight strongest edges with bootstrapped 95% confidence intervals (CI) in the baseline network of remitted patients and in the two post-treatment networks

Symptom 1	Symptom 2	Edge weight	95% CI
Baseline network, remitted patients			
Feeling inferior to others	Feeling of worthlessness	0.278	[0.233, 0.402]
Feeling lonely	Feeling lonely even when you are with people	0.269	[0.244, 0.462]
Feeling hopeless about the future	Feeling of worthlessness	0.211	[0.154, 0.333]
Trouble remembering things	Trouble concentrating	0.204	[0.172, 0.419]
Thoughts of ending your life	Thoughts about death and dying	0.203	[0.163, 0.461]
Feeling afraid in open spaces	Feeling afraid to travel on buses, subways, or trains	0.201	[0.159, 0.417]
Nervousness or shakiness inside	Feeling tense or keyed up	0.191	[0.155, 0.390]
Numbness or tingling in parts of your body	Feeling weak in parts of your body	0.167	[0.113, 0.413]
Post-treatment network, remitted patients			
Feeling lonely	Feeling lonely even when you are with people	0.515	[0.373, 0.567]
Trouble remembering things	Trouble concentrating	0.384	[0.244, 0.441]
Thoughts of ending your life	Thoughts about death and dying	0.362	[0.209, 0.434]
Having urges to beat, injure, or harm someone	Having urges to break or smash things	0.329	[0.162, 0.428]
Feeling afraid in open spaces	Feeling afraid to travel on buses, subways, or trains	0.298	[0.152, 0.391]
Feeling inferior to others	Feeling of worthlessness	0.269	[0.145, 0.341]
Nervousness or shakiness inside	Feeling tense or keyed up	0.261	[0.147, 0.336]
Feeling no interest in things	Feeling hopeless about the future	0.252	[0.126, 0.323]
Post-treatment network, non-remitted patients			

Feeling lonely	Feeling lonely even when you are with people	0.441	[0.306, 0.496]
Feeling inferior to others	Feeling of worthlessness	0.435	[0.322, 0.483]
Thoughts of ending your life	Thoughts about death and dying	0.403	[0.262, 0.466]
Trouble remembering things	Trouble concentrating	0.303	[0.190, 0.378]
Others not giving you proper credit for your achievements	Feeling that people will take advantage of you if you let them	0.299	[0.161, 0.373]
Feeling that people are unfriendly or dislike you	Feeling of being watched or talked about by others	0.294	[0.165, 0.370]
Having urges to beat, injure, or harm someone	Having urges to break or smash things	0.285	[0.128, 0.389]
Nervousness or shakiness inside	Feeling tense or keyed up	0.272	[0.143, 0.352]

Note. Edge weights are regularized partial correlations from the Gaussian Graphical Model (EBICglasso, Spearman). The eight strongest edges by absolute weight are shown for each network. CIs are based on 1000 nonparametric bootstrap resamples.

Table C2 Expected influence of all nodes before and after treatment in remitted and non-remitted patients

Node	EI pre	EI post	z pre	z post	Direction	p (FDR)
Remitted patients (n = 277)						
Nerv	0.276	0.810	+0.52	-0.65	decrease	0.032
Dizzi	0.022	0.853	-0.82	-0.40	increase	0.031
MindC	0.000	0.433	-0.94	-2.85	decrease	0.041
Blame	0.000	0.886	-0.94	-0.21	increase	0.006
MemP	0.204	0.913	+0.14	-0.05	decrease	0.009
Irrit	0.116	0.976	-0.33	+0.32	increase	0.013
PainChest	0.000	0.981	-0.94	+0.35	increase	0.005
FearOpen	0.379	1.088	+1.05	+0.97	decrease	0.016
SuiciT	0.203	0.789	+0.13	-0.77	decrease	0.018
Distrust	0.191	1.095	+0.07	+1.01	increase	0.009
NoApp	0.000	0.862	-0.94	-0.34	increase	< .001
FearSud	0.000	0.854	-0.94	-0.39	increase	0.016
Temper	0.155	1.235	-0.12	+1.83	increase	0.001
ALone	0.394	1.100	+1.13	+1.04	decrease	0.005
NoInitia	0.409	1.082	+1.21	+0.94	decrease	< .001
Lonely	0.451	0.896	+1.43	-0.15	decrease	0.021
Blue	0.410	1.025	+1.22	+0.60	decrease	0.003
Nointe	0.419	1.133	+1.26	+1.23	decrease	0.003
Fearful	0.178	1.013	-0.00	+0.53	increase	0.009
Hurt	0.289	1.202	+0.58	+1.64	increase	0.002
Dislike	0.397	1.082	+1.15	+0.94	decrease	0.009
Inferio	0.671	1.067	+2.59	+0.85	decrease	0.006
Nausea	0.046	0.883	-0.69	-0.23	increase	0.029
Watched	0.215	0.802	+0.19	-0.70	decrease	0.026
Sleep	0.000	0.569	-0.94	-2.06	decrease	0.036
Compul	0.000	0.624	-0.94	-1.73	decrease	0.025

Decision	0.068	0.915	-0.58	-0.04	increase	0.005
FearTran	0.338	0.845	+0.84	-0.44	decrease	0.031
Breath	0.000	0.772	-0.94	-0.87	increase	0.015
Flashes	0.000	0.846	-0.94	-0.44	increase	0.022
Avoid	0.117	0.834	-0.32	-0.51	decrease	0.021
Blank	0.076	0.691	-0.54	-1.34	decrease	0.014
Numb	0.168	0.642	-0.06	-1.63	decrease	0.036
Punish	0.000	0.717	-0.94	-1.19	decrease	0.034
Nohope	0.446	1.243	+1.41	+1.88	increase	< .001
ConcenP	0.373	1.190	+1.02	+1.57	increase	0.003
Weak	0.169	1.017	-0.05	+0.56	increase	0.031
Tension	0.264	0.993	+0.45	+0.42	decrease	0.011
DeathT	0.203	1.000	+0.13	+0.46	increase	0.014
Violence	0.026	0.805	-0.80	-0.68	increase	0.006
Break	0.026	0.866	-0.80	-0.33	increase	0.011
Shy	0.061	0.924	-0.61	+0.01	increase	0.004
FearCro	0.293	0.920	+0.60	-0.01	decrease	0.016
NotClose	0.052	0.833	-0.67	-0.51	increase	0.008
Panic	0.031	0.902	-0.77	-0.12	increase	0.022
Argue	0.000	1.032	-0.94	+0.65	increase	0.005
FearAlo	0.000	0.718	-0.94	-1.19	decrease	0.032
Nocredit	0.128	0.858	-0.27	-0.37	decrease	0.017
Restless	0.000	0.802	-0.94	-0.69	increase	0.017
NoWorth	0.859	1.165	+3.58	+1.42	decrease	0.017
Exploit	0.228	1.102	+0.26	+1.05	increase	0.014
Guilt	0.065	0.934	-0.60	+0.07	increase	0.003
MindP	0.041	1.011	-0.72	+0.52	increase	0.003
Non-remitted patients (n = 266)						
Nerv	0.076	0.971	-0.16	+0.30	increase	0.160
Dizzi	0.000	0.836	-0.70	-0.53	increase	0.280

MindC	0.000	0.788	-0.70	-0.83	decrease	0.067
Blame	0.021	0.708	-0.56	-1.32	decrease	0.160
MemP	0.163	0.836	+0.47	-0.53	decrease	0.280
Irrit	0.000	0.945	-0.70	+0.14	increase	0.193
PainChest	0.000	0.686	-0.70	-1.46	decrease	0.362
FearOpen	0.251	0.904	+1.09	-0.11	decrease	0.305
SuiciT	0.106	0.825	+0.06	-0.60	decrease	0.160
Distrust	0.181	1.249	+0.60	+2.02	increase	0.160
NoApp	0.000	0.722	-0.70	-1.24	decrease	0.039
FearSud	0.049	1.046	-0.36	+0.76	increase	0.080
Temper	0.020	0.859	-0.56	-0.39	increase	0.170
ALone	0.430	1.079	+2.38	+0.97	decrease	0.280
NoInitia	0.224	0.941	+0.90	+0.12	decrease	0.080
Lonely	0.328	0.933	+1.65	+0.07	decrease	0.157
Blue	0.137	1.132	+0.28	+1.29	increase	0.050
Nointe	0.174	0.959	+0.54	+0.23	decrease	0.080
Fearful	0.003	0.969	-0.69	+0.29	increase	0.199
Hurt	0.020	1.194	-0.56	+1.68	increase	0.080
Dislike	0.320	0.987	+1.59	+0.40	decrease	0.193
Inferio	0.365	1.036	+1.92	+0.70	decrease	0.097
Nausea	0.000	0.878	-0.70	-0.27	increase	0.280
Watched	0.167	0.929	+0.49	+0.04	decrease	0.305
Sleep	0.000	0.551	-0.70	-2.29	decrease	0.305
Compul	0.000	0.469	-0.70	-2.80	decrease	0.469
Decision	0.000	1.217	-0.70	+1.82	increase	0.021
FearTran	0.251	0.967	+1.09	+0.28	decrease	0.280
Breath	0.000	0.896	-0.70	-0.17	increase	0.280
Flashes	0.000	0.936	-0.70	+0.09	increase	0.280
Avoid	0.000	0.825	-0.70	-0.60	increase	0.280
Blank	0.000	0.996	-0.70	+0.45	increase	0.039
Numb	0.016	0.714	-0.59	-1.29	decrease	0.336

Punish	0.000	0.682	-0.70	-1.48	decrease	0.280
Nohope	0.310	1.102	+1.52	+1.11	decrease	0.080
ConcenP	0.181	1.140	+0.60	+1.35	increase	0.153
Weak	0.016	1.124	-0.59	+1.25	increase	0.157
Tension	0.076	0.992	-0.16	+0.43	increase	0.164
DeathT	0.106	0.882	+0.06	-0.25	decrease	0.164
Violence	0.027	0.695	-0.51	-1.40	decrease	0.157
Break	0.027	0.829	-0.51	-0.58	decrease	0.305
Shy	0.000	0.828	-0.70	-0.58	increase	0.199
FearCro	0.188	1.022	+0.64	+0.61	decrease	0.160
NotClose	0.000	0.847	-0.70	-0.47	increase	0.089
Panic	0.051	0.996	-0.34	+0.46	increase	0.280
Argue	0.000	0.873	-0.70	-0.31	increase	0.160
FearAlo	0.000	0.908	-0.70	-0.09	increase	0.157
Nocredit	0.010	1.040	-0.63	+0.73	increase	0.067
Restless	0.000	0.880	-0.70	-0.26	increase	0.164
NoWorth	0.666	1.179	+4.08	+1.59	decrease	0.157
Exploit	0.137	0.817	+0.28	-0.65	decrease	0.276
Guilt	0.109	1.022	+0.07	+0.62	increase	0.160
MindP	0.000	1.040	-0.70	+0.73	increase	0.184

Note. EI = expected influence, the sum of the edge weights connected to a node. Raw EI values are shown together with the standardized values (z) across the 53 nodes within the baseline network of remitted patients and in the two post-treatment networks, as displayed in Figure 4. Direction refers to the change in standardized expected influence from pre- to post-treatment. p values are from the dependent (paired) permutation network comparison test with 10,000 permutations, adjusted with the False Discovery Rate across the 53 nodes.

Table C3 Descriptive statistics of the individual symptoms discussed in the text

Node	Symptom	Remitted pre	Remitted post	Non-remitted pre	Non-remitted post
Nerv	Nervousness or shakiness inside	1.81 (1.11)	1.00 (0.89)	1.70 (1.08)	1.12 (0.98)
Dizzi	Faintness or dizziness	0.60 (0.88)	0.31 (0.60)	0.61 (0.87)	0.36 (0.70)
MemP	Trouble remembering things	1.42 (1.12)	0.95 (1.07)	1.43 (1.13)	1.08 (1.03)
Irrit	Feeling easily annoyed or irritated	1.54 (1.13)	0.98 (1.01)	1.51 (1.12)	1.09 (1.00)
FearOpen	Feeling afraid in open spaces	0.71 (1.02)	0.40 (0.83)	0.82 (1.08)	0.51 (0.78)
SuiciT	Thoughts of ending your life	0.29 (0.73)	0.13 (0.45)	0.38 (0.74)	0.23 (0.60)
Distrust	Feeling that most people cannot be trusted	1.04 (1.15)	0.60 (0.89)	1.09 (1.14)	0.70 (0.91)
Temper	Temper outbursts that you could not control	1.38 (1.23)	0.84 (1.02)	1.15 (1.09)	0.79 (0.95)
ALone	Feeling lonely even when you are with people	1.52 (1.18)	0.79 (1.00)	1.52 (1.24)	1.03 (1.12)
Lonely	Feeling lonely	1.75 (1.22)	0.98 (1.14)	1.71 (1.26)	1.18 (1.09)
Inferio	Feeling inferior to others	2.14 (1.21)	1.09 (1.11)	2.20 (1.22)	1.39 (1.13)
Watched	Feeling of being watched or talked about by others	1.33 (1.28)	0.53 (0.84)	1.53 (1.34)	0.74 (0.88)
Decision	Difficulty making decisions	1.80 (1.21)	1.10 (1.07)	1.98 (1.13)	1.30 (1.10)
FearTran	Feeling afraid to travel on buses, subways, or trains	0.64 (1.04)	0.36 (0.78)	0.72 (1.14)	0.44 (0.85)
Nohope	Feeling hopeless about the future	1.74 (1.22)	0.80 (1.05)	1.89 (1.29)	1.08 (1.16)
ConcenP	Trouble concentrating	2.01 (1.23)	1.22 (1.18)	1.97 (1.24)	1.45 (1.10)
Tension	Feeling tense or	1.77 (1.23)	1.13 (1.00)	1.77 (1.14)	1.29 (0.99)

	keyed up				
FearCro	Feeling uneasy in crowds	1.31 (1.29)	0.83 (1.08)	1.55 (1.36)	1.00 (1.12)
Argue	Getting into frequent arguments	0.85 (1.11)	0.45 (0.85)	0.79 (1.09)	0.53 (0.92)
NoWorth	Feeling of worthlessness	1.81 (1.33)	0.75 (1.11)	1.97 (1.32)	1.03 (1.19)
MindP	The idea that something is wrong with your mind	1.19 (1.24)	0.54 (0.91)	1.36 (1.29)	0.75 (1.07)

Note. Values are means (standard deviations) on the 0–4 scale of the Brief Symptom Inventory. Node labels follow Table 2.
