# Supplementary material for: Network changes in psychopathological symptoms following cognitive behavioral therapy for social anxiety disorder: evidence from 29 university outpatient clinics
Source: BMC Psychol. 2026 Nov 10;14:1627. doi: 10.1186/s40359-026-05900-9 (PMC13750052; doi:10.1186/s40359-026-05900-9)
Supplement: Supplementary file 2 [file 40359_2026_5900_MOESM2_ESM.pdf]

Appendix A. Supplementary Material: Bootstrapped stability test figures

Note. Each figure displays the edge weights of one network together with their bootstrap confidence intervals (1000 nonparametric bootstrap samples). Edges are ordered from the lowest to the highest weight. Red dots represent the sample edge weights, black dots the means of the bootstrap samples, and the grey bands the 95% confidence intervals. In all six networks, edges with medium and strong weights were estimated with narrow confidence intervals, and the confidence intervals of the strongest edges did not include zero, which supports the interpretation of these edges. Weaker edges varied more across bootstrap samples and should therefore not be interpreted individually. Together with the centrality stability coefficient reported in the main text, these results indicate that the network structure can be interpreted with confidence for medium and strong edges, whereas the centrality estimates, and in particular the estimates after treatment, should be interpreted with caution.

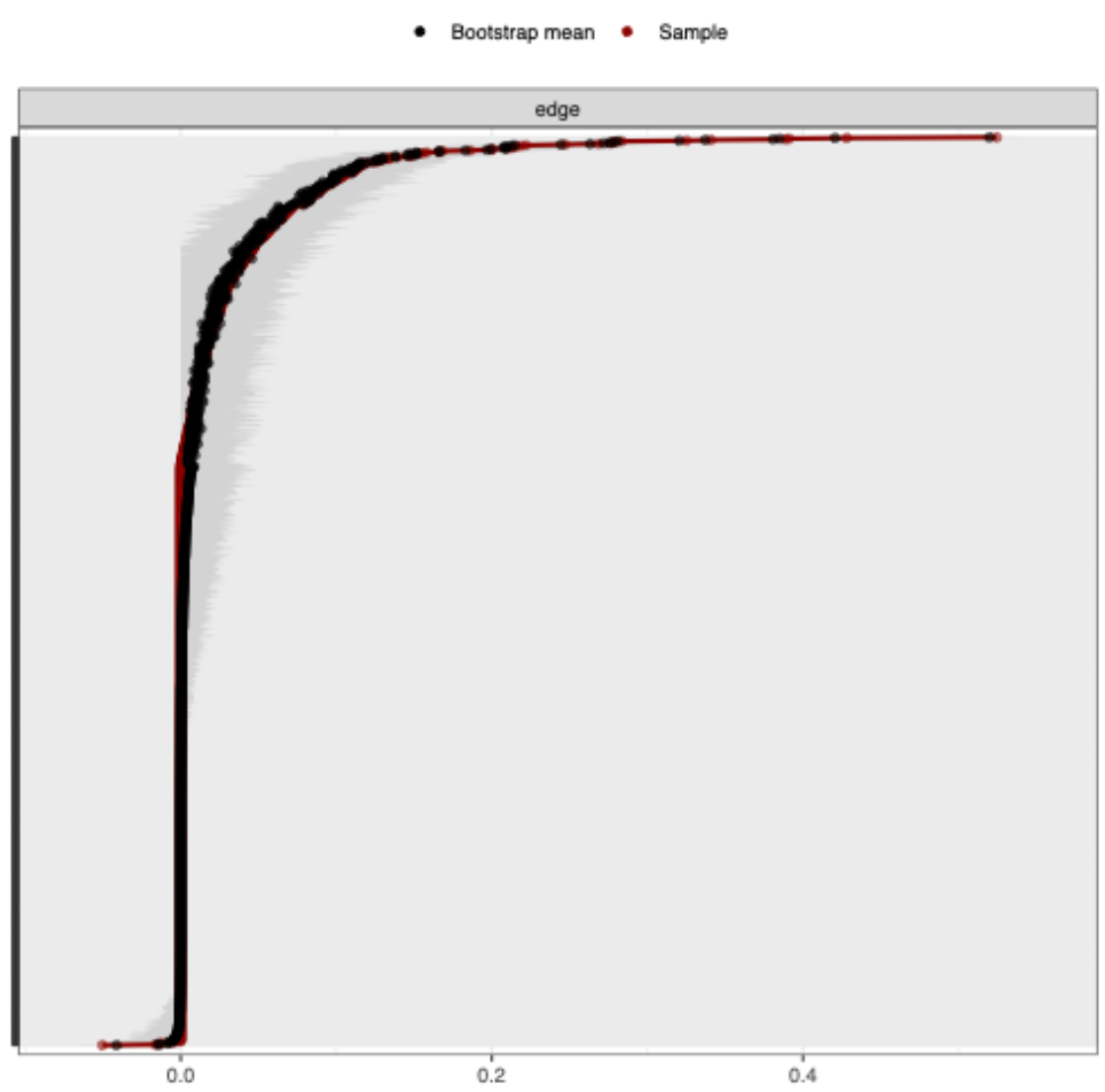

Supplementary Figure A.1.

Bootstrapped stability test figures of baseline network for patients with SAD.

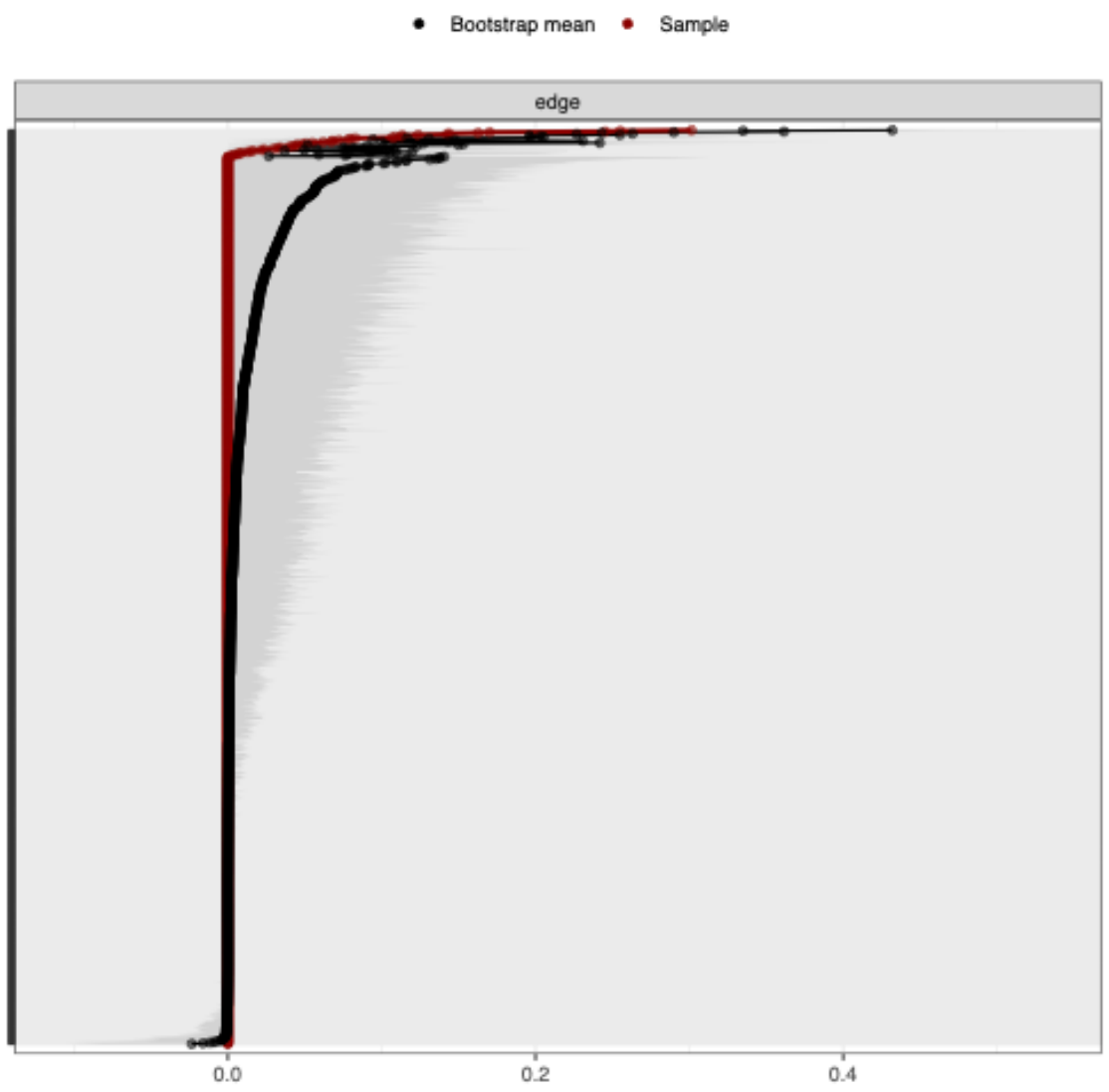

Supplementary Figure A.2.

Bootstrapped stability test figures of baseline network for patients with SAD without additional comorbidity.

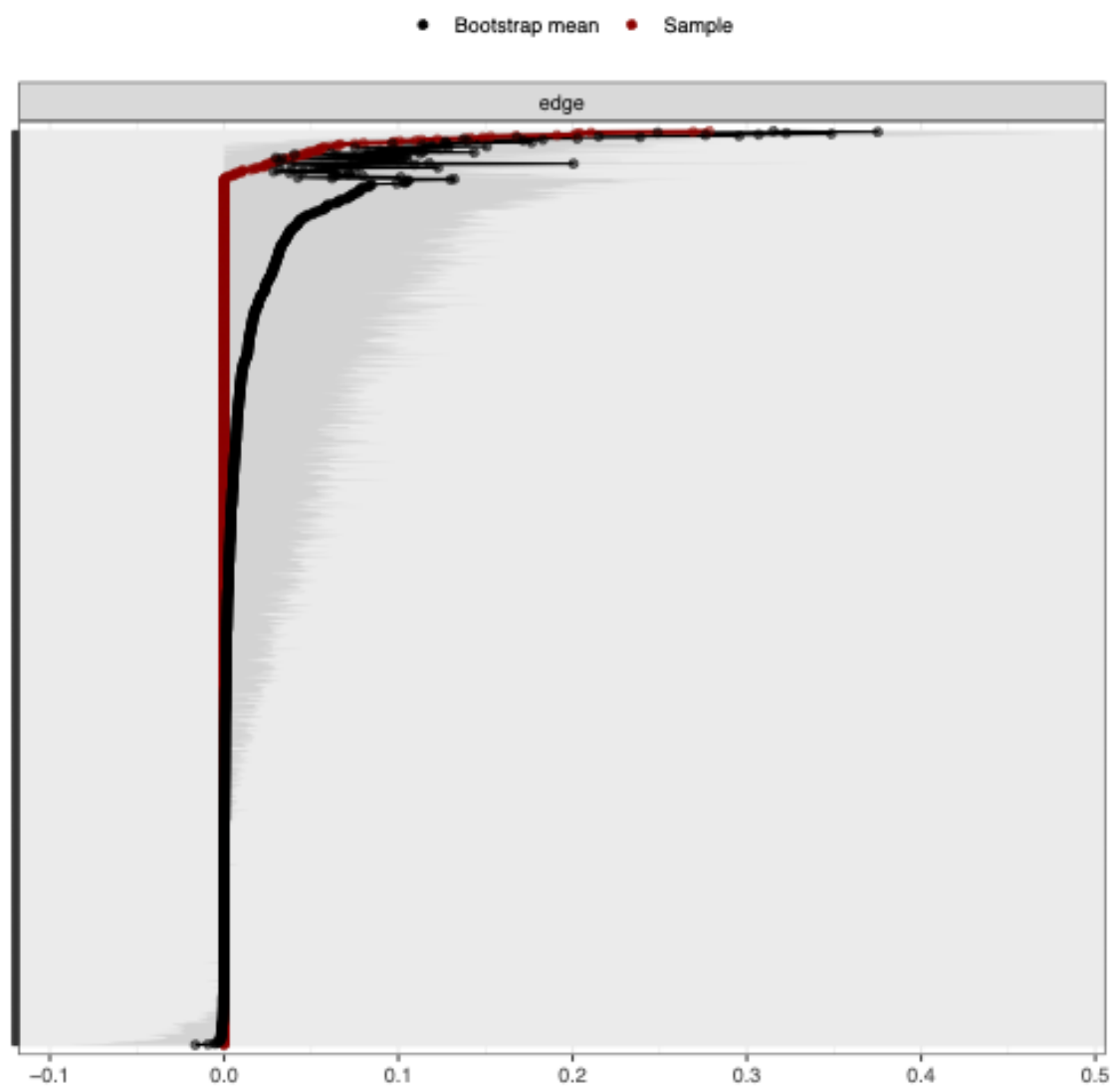

Supplementary Figure A.3.

Bootstrapped stability test figures of baseline network for patients who achieve remission from social anxiety disorder at post-treatment.

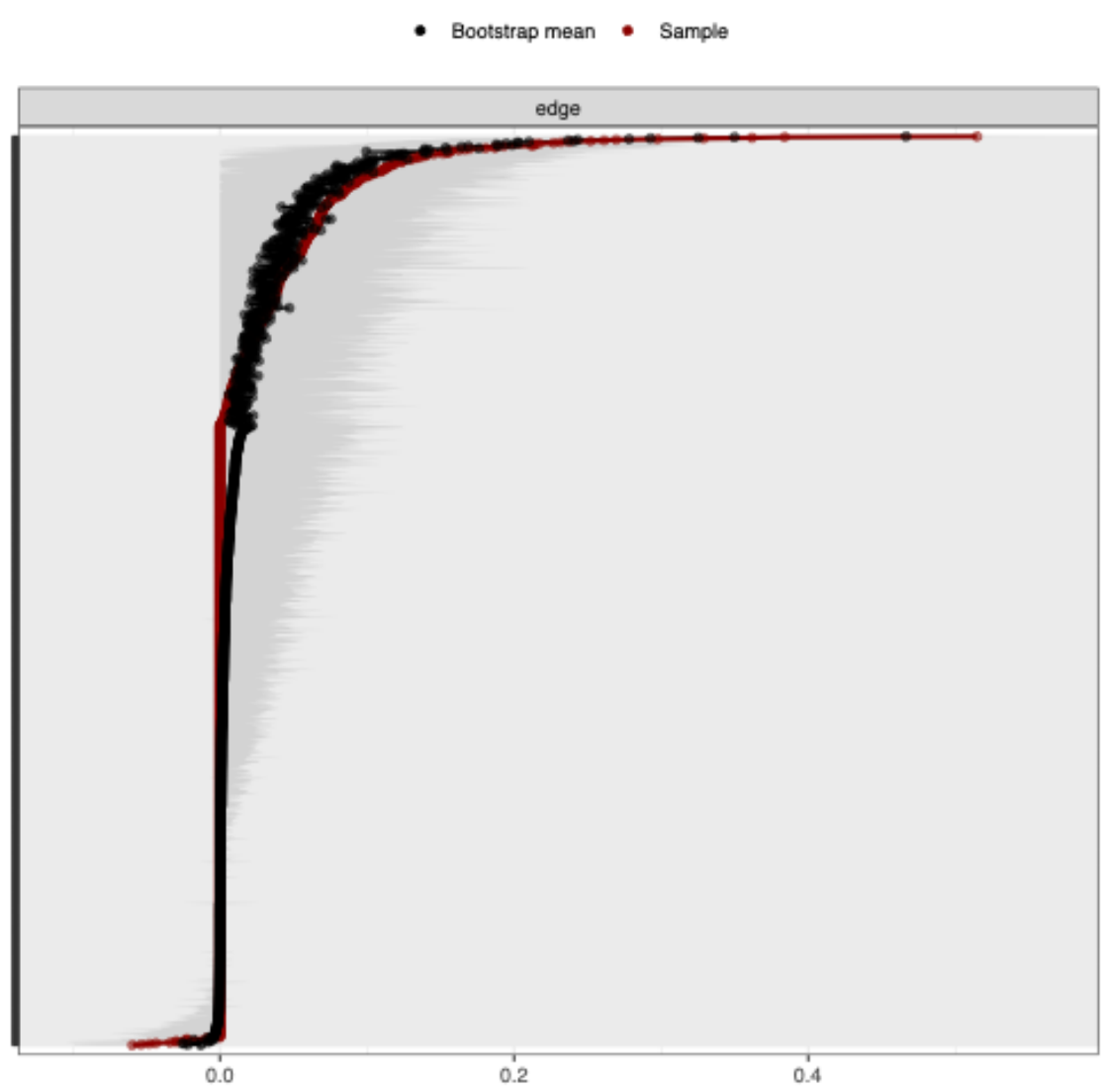

Supplementary Figure A.4.

Bootstrapped stability test figures of post-treatment network for remitted patients.

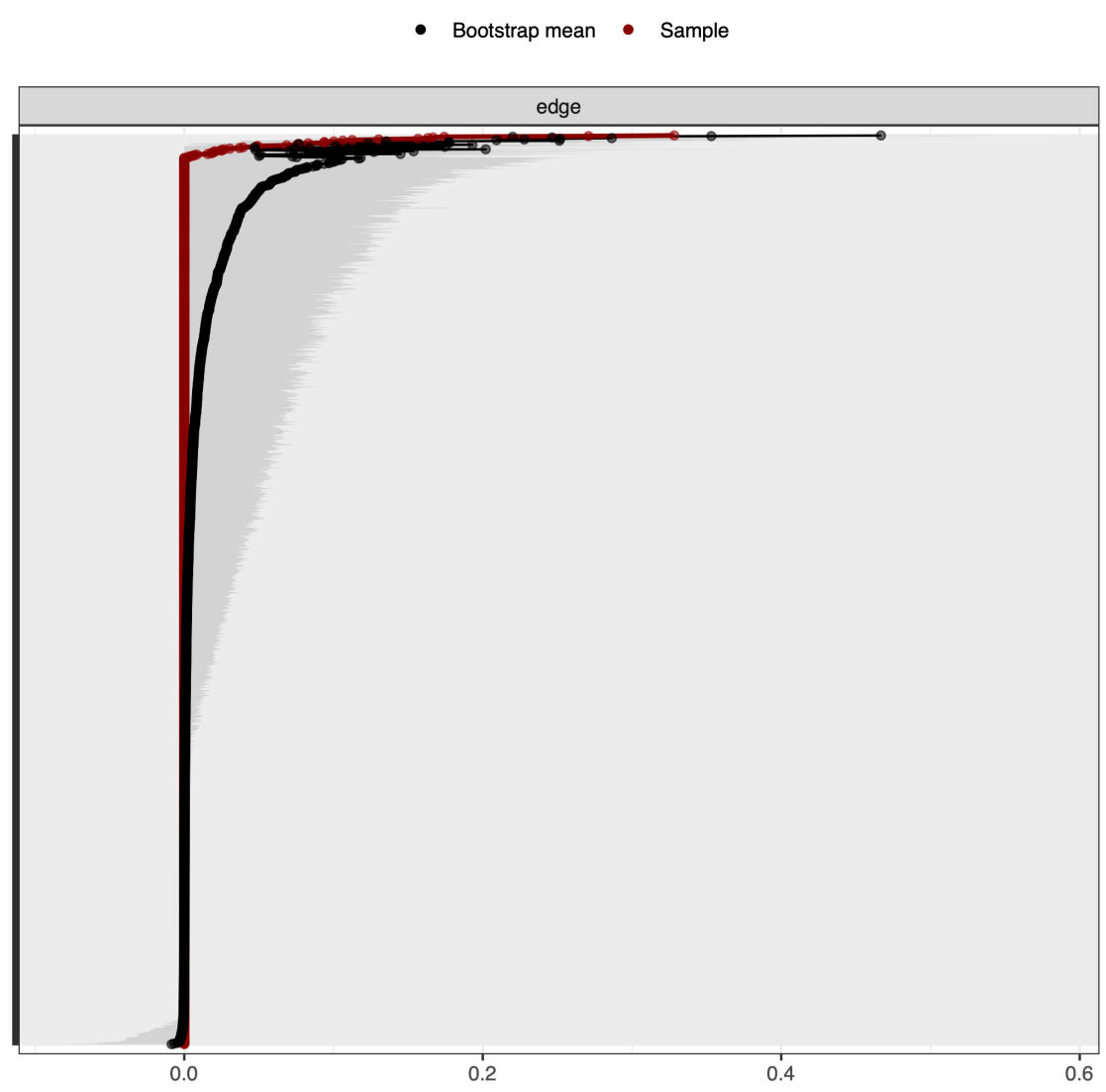

Supplementary Figure A.5.

Bootstrapped stability test figures of baseline network for patients who do not achieve remission from social anxiety disorder at post-treatment.

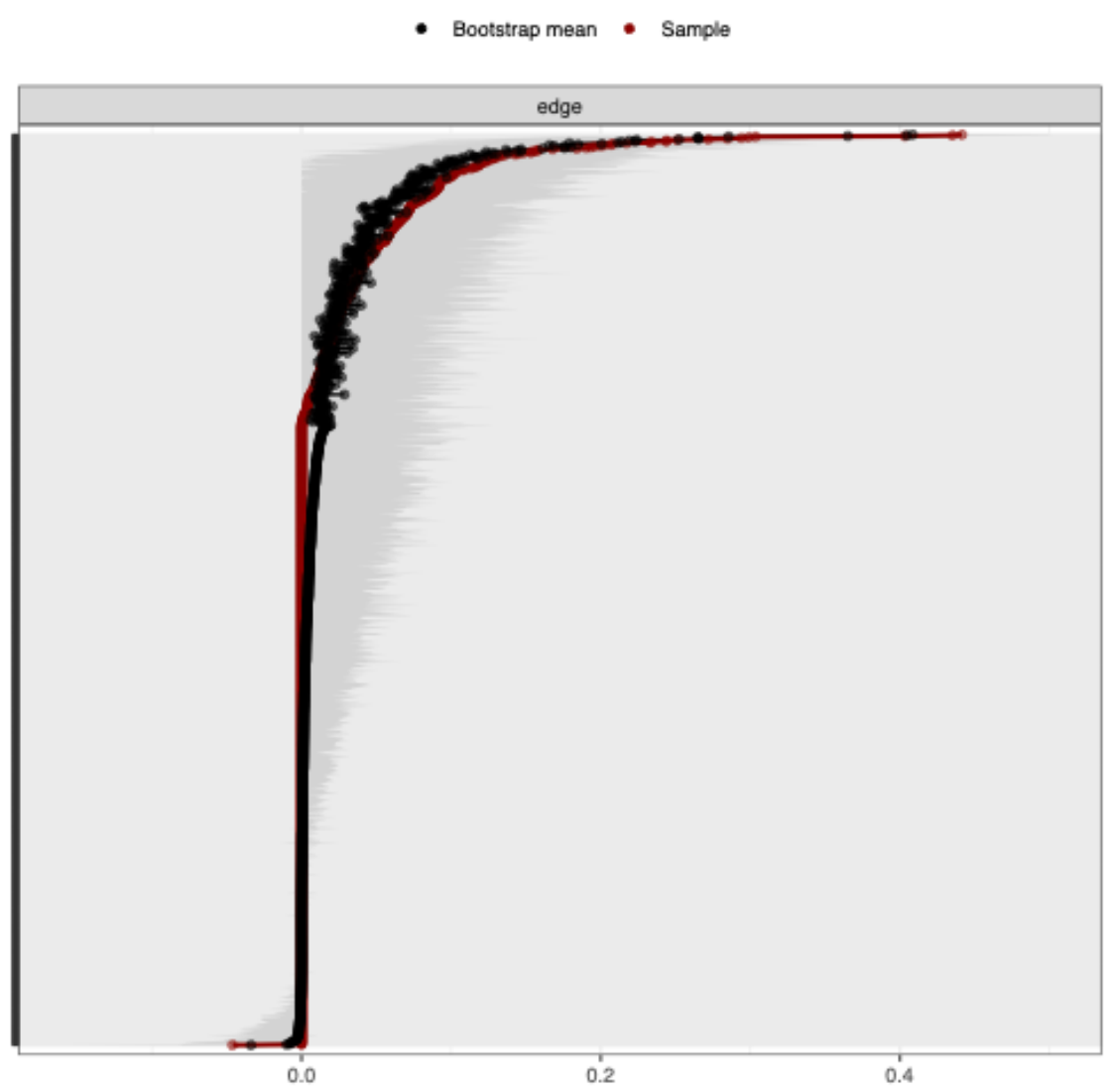

Supplementary Figure A.6.

Bootstrapped stability test figures of post-treatment network for non-remitted patients.
