# Supplementary material for: Network changes in psychopathological symptoms following cognitive behavioral therapy for social anxiety disorder: evidence from 29 university outpatient clinics
Source: BMC Psychol. 2026 Nov 10;14:1627. doi: 10.1186/s40359-026-05900-9 (PMC13750052; doi:10.1186/s40359-026-05900-9)
Supplement: Supplementary file 3 [file 40359_2026_5900_MOESM3_ESM.pdf]

The following six Figures are in sequence:

Figure B.1. Bootstrapped edge difference test figures of baseline network for patients with SAD;

Figure B.2. Bootstrapped edge difference test figures of baseline network for patients without additional comorbidity;

Figure B.3. Bootstrapped edge difference test figures of baseline network for patients who achieve remission from social anxiety disorder at post-treatment;

Figure B.4. Bootstrapped edge difference test figures of post-treatment network for remitted patients;

Figure B.5. Bootstrapped edge difference test figures of baseline network for patients who do not achieve remission from social anxiety disorder at post-treatment;

Figure B.6. Bootstrapped edge difference test figures of post-treatment network for non-remitted patients.

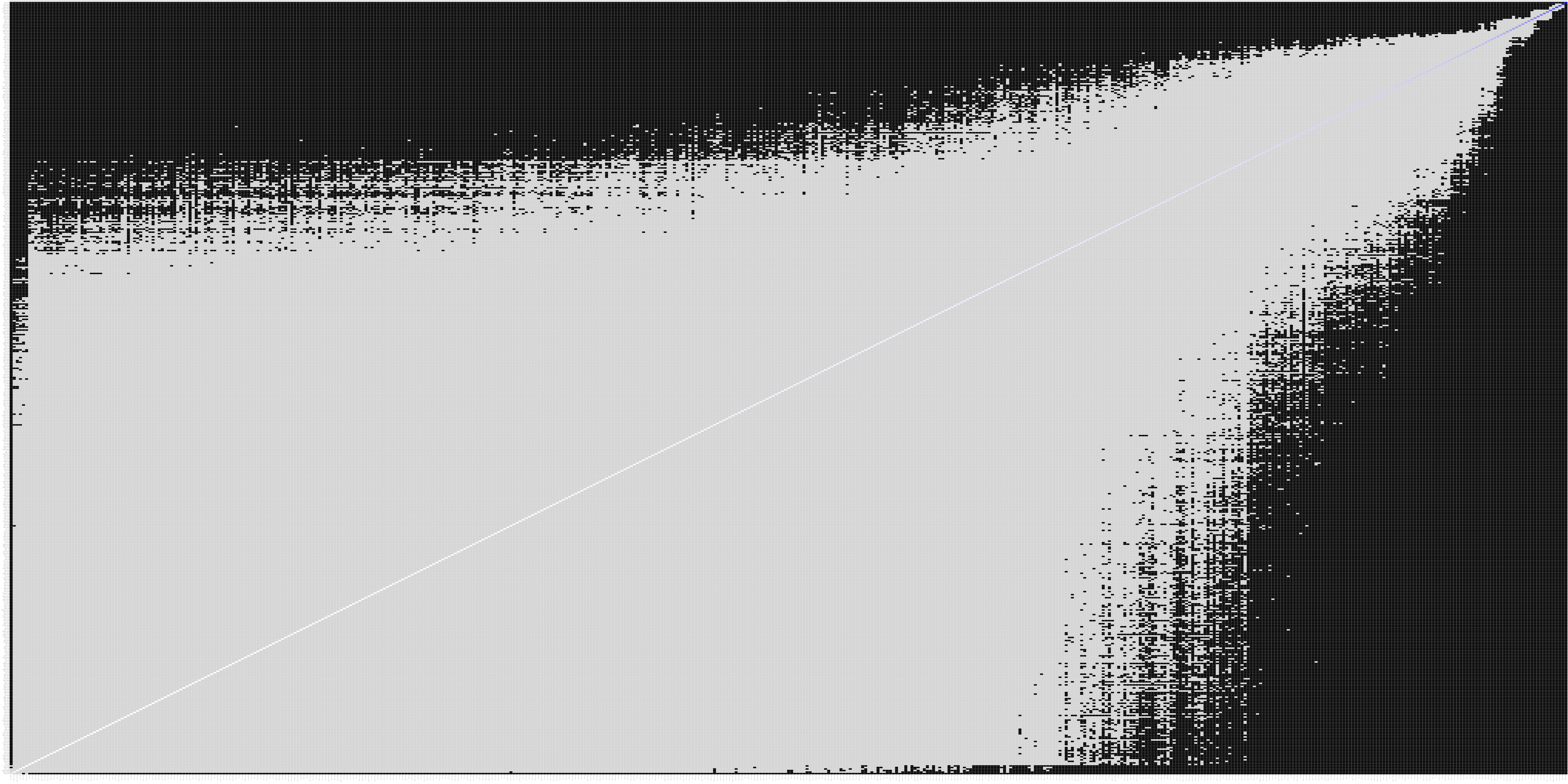

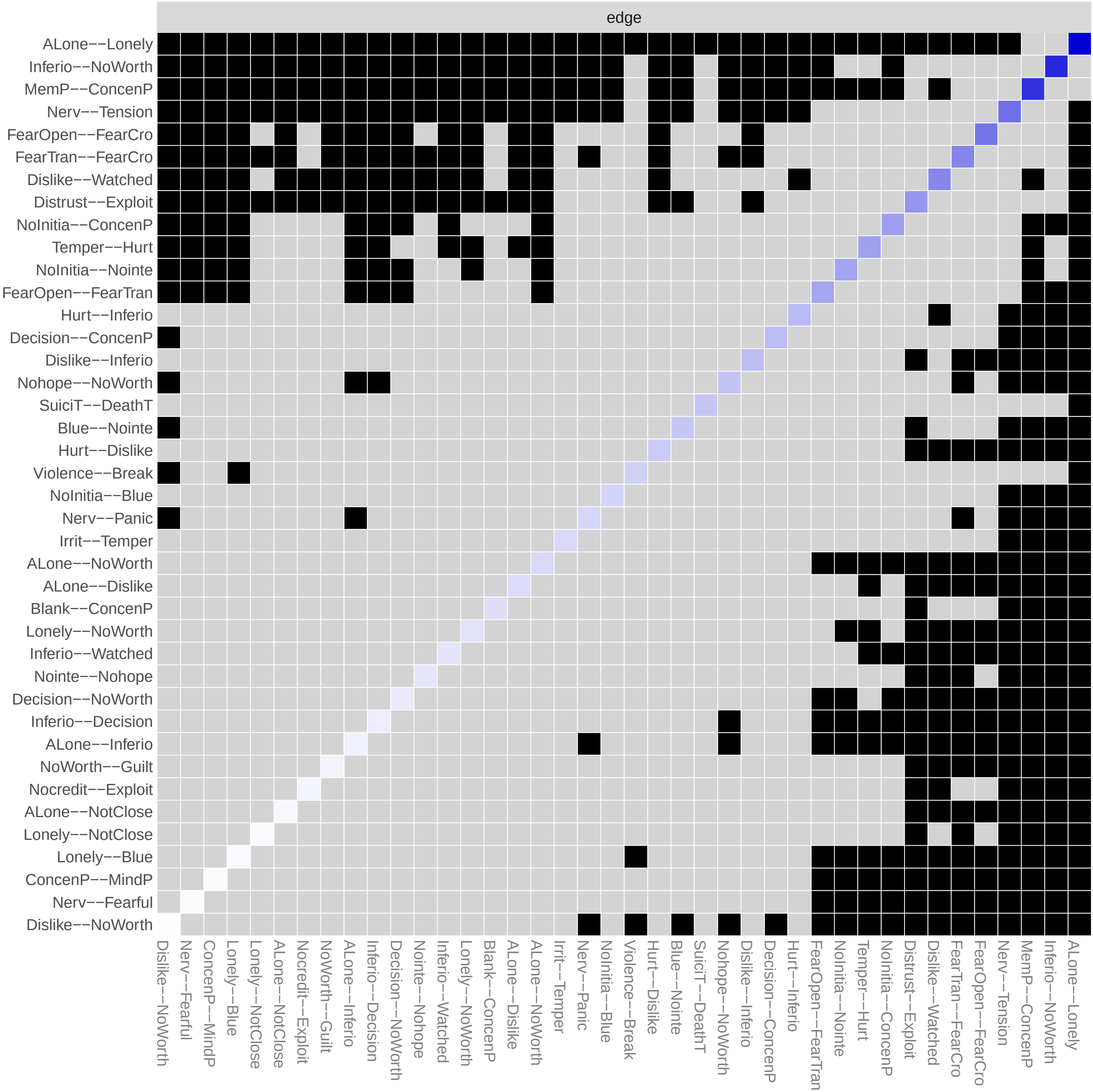
edge
ALone--Lonely
Inferio--NoWorth
MemP--ConcenP
Nerv--Tension
FearOpen--FearCro
FearTran--FearCro
Dislike--Watched
Distrust--Exploit
NoInitia--ConcenP
Temper--Hurt
NoInitia--Nointe
FearOpen--FearTran
Hurt--Inferio
Decision--ConcenP
Dislike--Inferio
Nohope--NoWorth
SuiciT--DeathT
Blue--Nointe
Hurt--Dislike
Violence--Break
NoInitia--Blue
Nerv--Panic
Irrit--Temper
ALone--NoWorth
ALone--Dislike
Blank--ConcenP
Lonely--NoWorth
Inferio--Watched
Nointe--Nohope
Decision--NoWorth
Inferio--Decision
ALone--Inferio
NoWorth--Guilt
Nocredit--Exploit
ALone--NotClose
Lonely--NotClose
Lonely--Blue
ConcenP--MindP
Nerv--Fearful
Dislike--NoWorth
Dislike--NoWorth
Nerv--Fearful
ConcenP--MindP
Lonely--Blue
Lonely--NotClose
ALone--NotClose
Nocredit--Exploit
NoWorth--Guilt
ALone--Inferio
Inferio--Decision
Decision--NoWorth
Nointe--Nohope
Inferio--Watched
Lonely--NoWorth
Blank--ConcenP
ALone--Dislike
ALone--NoWorth
Irrit--Temper
Nerv--Panic
NoInitia--Blue
Violence--Break
Hurt--Dislike
Blue--Nointe
SuiciT--DeathT
Nohope--NoWorth
Dislike--Inferio
Decision--ConcenP
Hurt--Inferio
FearOpen--FearTran
NoInitia--Nointe
Temper--Hurt
NoInitia--ConcenP
Distrust--Exploit
Dislike--Watched
FearTran--FearCro
FearOpen--FearCro
Nerv--Tension
MemP--ConcenP
Inferio--NoWorth
ALone--Lonely

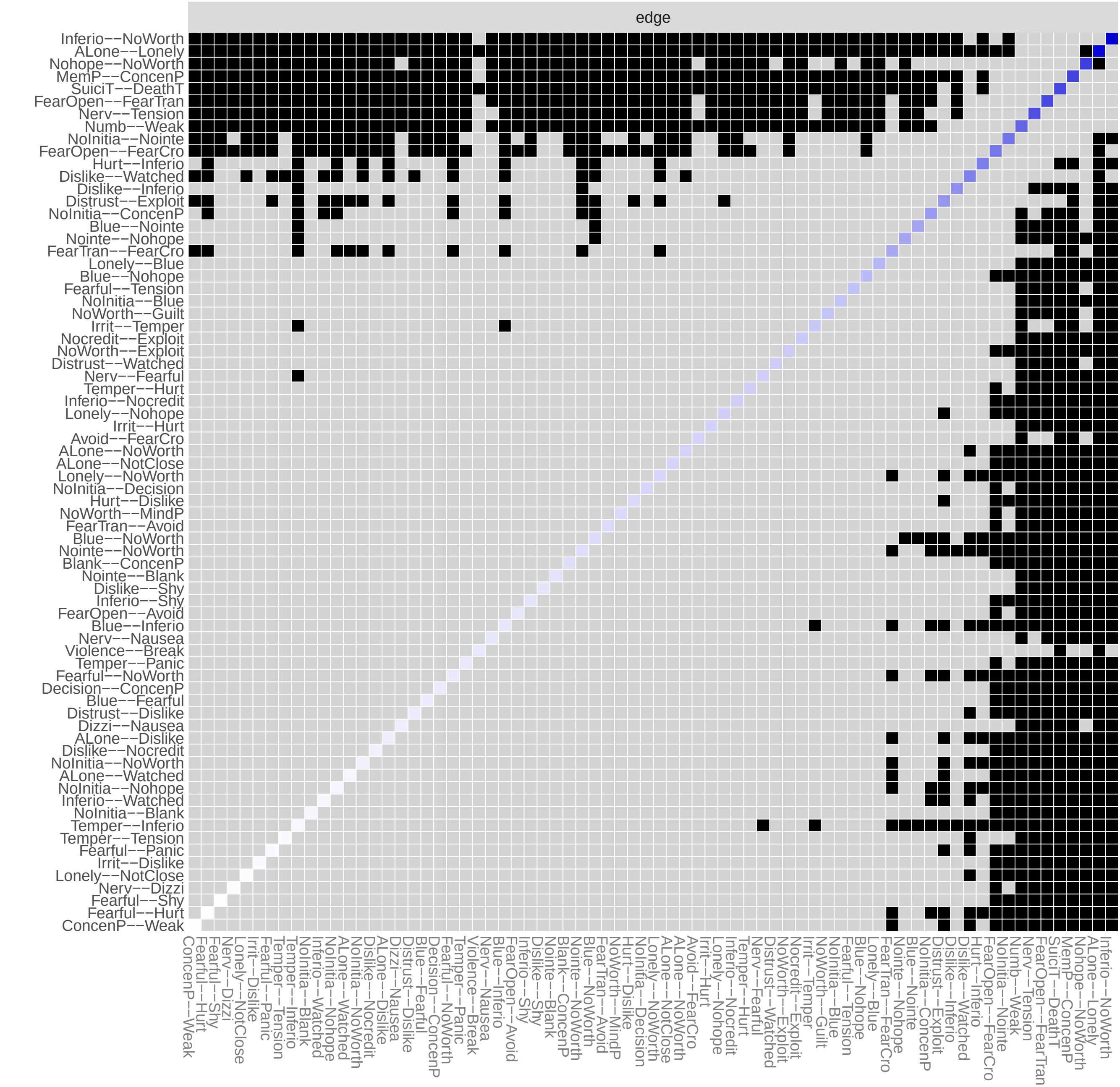
edge
Inferio--NoWorth
ALone--Lonely
Nohope--NoWorth
MemP--ConcenP
SuiciT--DeathT
FearOpen--FearTran
Nerv--Tension
Numb--Weak
NoInitia--Nointe
FearOpen--FearCro
Hurt--Inferio
Dislike--Watched
Dislike--Inferio
Distrust--Exploit
NoInitia--ConcenP
Blue--Nointe
Nointe--Nohope
FearTran--FearCro
Lonely--Blue
Blue--Nohope
Fearful--Tension
NoInitia--Blue
NoWorth--Guilt
Irrit--Temper
Nocredit--Exploit
NoWorth--Exploit
Distrust--Watched
Nerv--Fearful
Temper--Hurt
Inferio--Nocredit
Lonely--Nohope
Irrit--Hurt
Avoid--FearCro
ALone--NoWorth
ALone--NotClose
Lonely--NoWorth
NoInitia--Decision
Hurt--Dislike
NoWorth--MindP
FearTran--Avoid
Blue--NoWorth
Nointe--NoWorth
Blank--ConcenP
Nointe--Blank
Dislike--Shy
Inferio--Shy
FearOpen--Avoid
Blue--Inferio
Nerv--Nausea
Violence--Break
Temper--Panic
Fearful--NoWorth
Decision--ConcenP
Blue--Fearful
Distrust--Dislike
Dizzi--Nausea
ALone--Dislike
Dislike--Nocredit
NoInitia--NoWorth
ALone--Watched
NoInitia--Nohope
Inferio--Watched
NoInitia--Blank
Temper--Inferio
Temper--Tension
Fearful--Panic
Irrit--Dislike
Lonely--NotClose
Nerv--Dizzi
Fearful--Shy
Fearful--Hurt
ConcenP--Weak
ConcenP--Weak
Fearful--Hurt
Fearful--Shy
Nerv--Dizzi
Lonely--NotClose
Irrit--Dislike
Fearful--Panic
Temper--Tension
Temper--Inferio
NoInitia--Blank
Inferio--Watched
NoInitia--Nohope
ALone--Watched
NoInitia--NoWorth
Dislike--Nocredit
ALone--Dislike
Dizzi--Nausea
Distrust--Dislike
Blue--Fearful
Decision--ConcenP
Fearful--NoWorth
Temper--Panic
Violence--Break
Nerv--Nausea
Blue--Inferio
FearOpen--Avoid
Inferio--Shy
Dislike--Shy
Nointe--Blank
Blank--ConcenP
Nointe--NoWorth
Blue--NoWorth
FearTran--Avoid
NoWorth--MindP
Hurt--Dislike
NoInitia--Decision
Lonely--NoWorth
ALone--NotClose
ALone--NoWorth
Avoid--FearCro
Irrit--Hurt
Lonely--Nohope
Inferio--Nocredit
Temper--Hurt
Nerv--Fearful
Distrust--Watched
NoWorth--Exploit
Nocredit--Exploit
Irrit--Temper
NoWorth--Guilt
NoInitia--Blue
Fearful--Tension
Blue--Nohope
Lonely--Blue
FearTran--FearCro
Nointe--Nohope
Blue--Nointe
NoInitia--ConcenP
Distrust--Exploit
Dislike--Inferio
Dislike--Watched
Hurt--Inferio
FearOpen--FearCro
NoInitia--Nointe
Numb--Weak
Nerv--Tension
FearOpen--FearTran
SuiciT--DeathT
MemP--ConcenP
Nohope--NoWorth
ALone--Lonely
Inferio--NoWorth

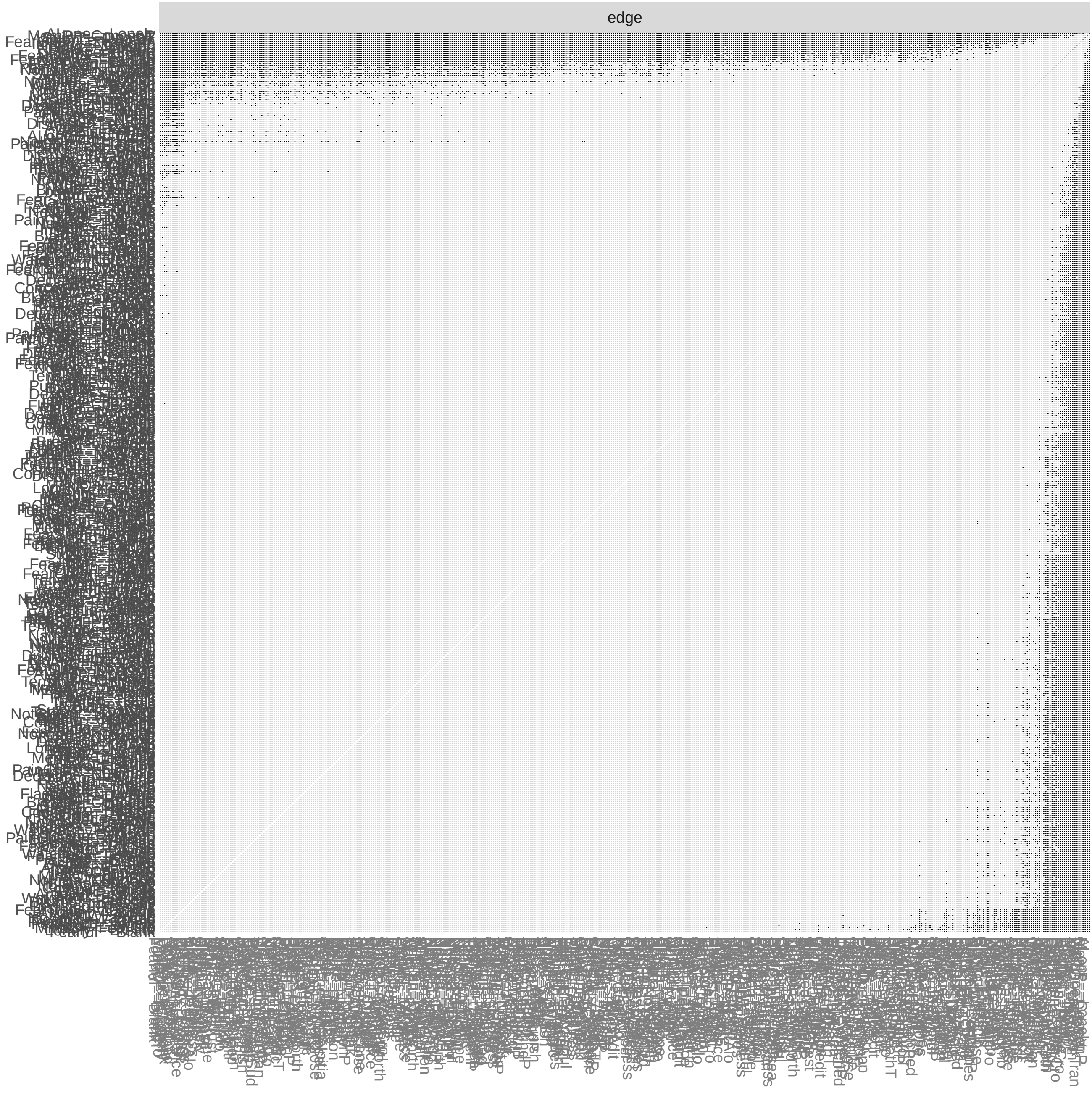
edge

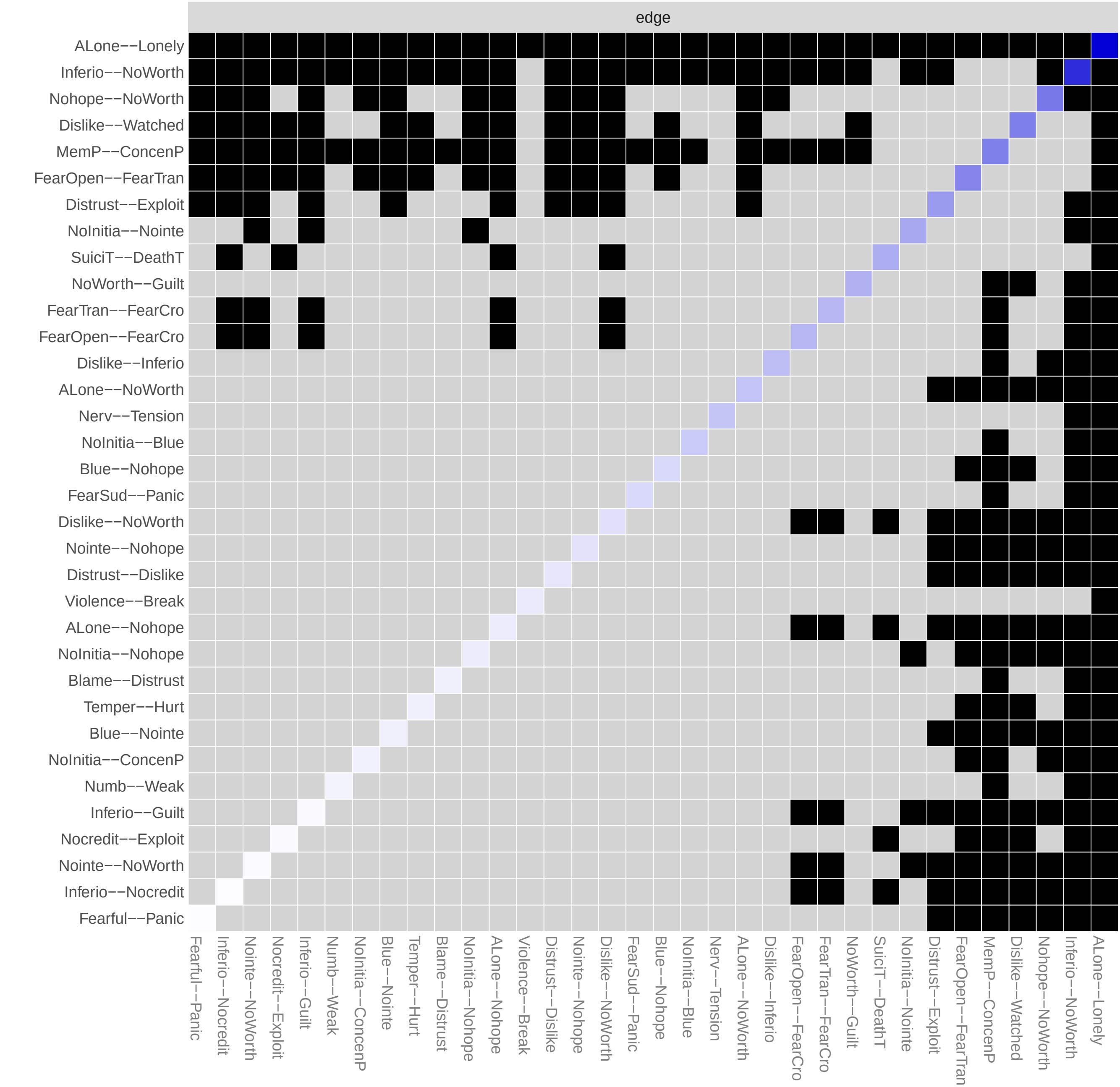
edge
ALone−−Lonely
Inferio−−NoWorth
Nohope−−NoWorth
Dislike−−Watched
MemP−−ConcenP
FearOpen−−FearTran
Distrust−−Exploit
NoInitia−−Nointe
SuiciT−−DeathT
NoWorth−−Guilt
FearTran−−FearCro
FearOpen−−FearCro
Dislike−−Inferio
ALone−−NoWorth
Nerv−−Tension
NoInitia−−Blue
Blue−−Nohope
FearSud−−Panic
Dislike−−NoWorth
Nointe−−Nohope
Distrust−−Dislike
Violence−−Break
ALone−−Nohope
NoInitia−−Nohope
Blame−−Distrust
Temper−−Hurt
Blue−−Nointe
NoInitia−−ConcenP
Numb−−Weak
Inferio−−Guilt
Nocredit−−Exploit
Nointe−−NoWorth
Inferio−−Nocredit
Fearful−−Panic

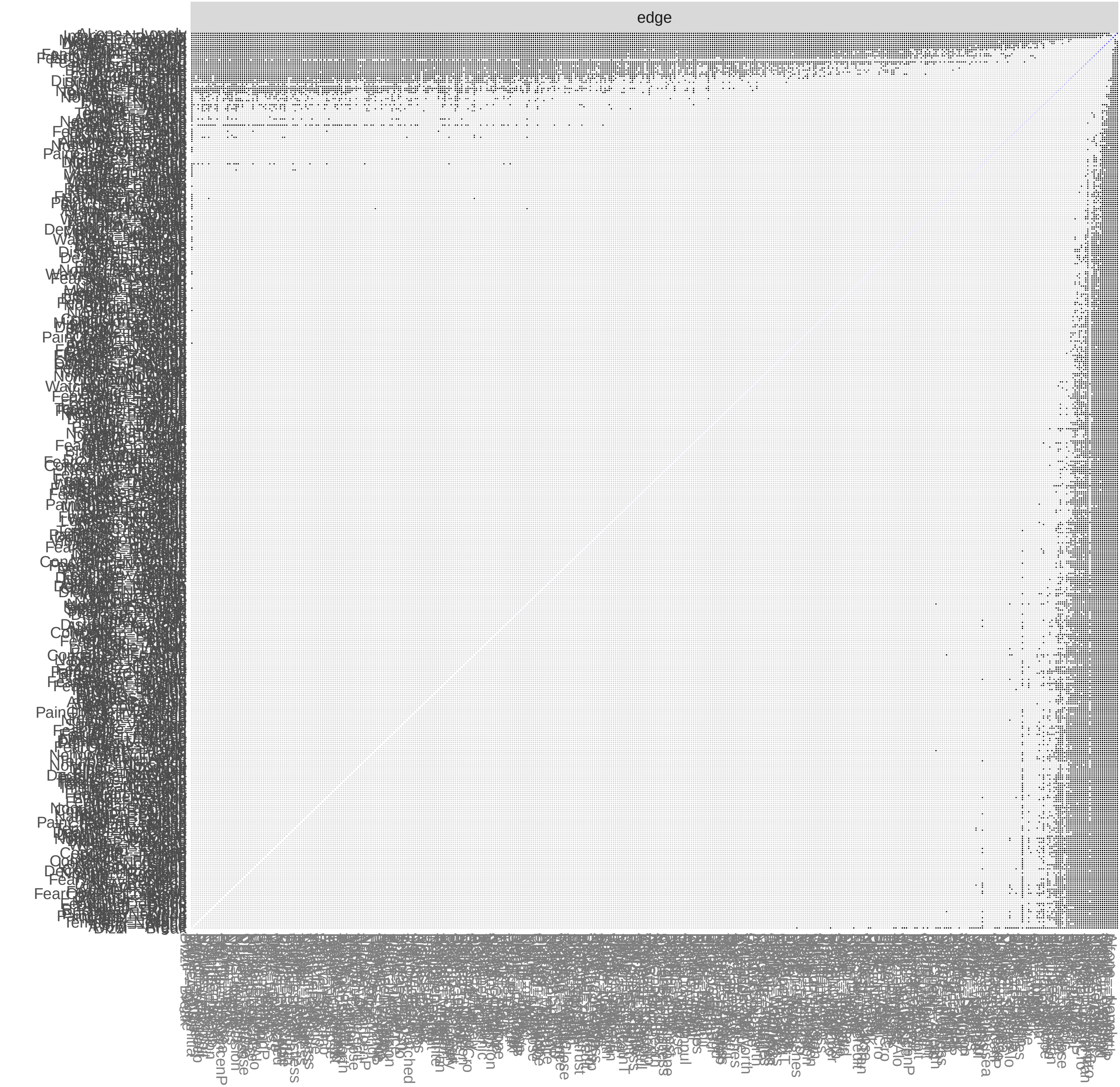
edge
